# Organic Amendments Alter Soil Hydrology and Belowground Microbiome of Tomato (*Solanum lycopersicum*)

**DOI:** 10.3390/microorganisms9081561

**Published:** 2021-07-22

**Authors:** Taylor Readyhough, Deborah A. Neher, Tucker Andrews

**Affiliations:** Department of Plant and Soil Science, University of Vermont, Jeffords Hall, Burlington, VT 05405, USA; treadyhough@gmail.com (T.R.); tucker.andrews@uvm.edu (T.A.)

**Keywords:** compost amendment, dairy manure compost, microbial community, poultry pellets, vermicompost, water holding capacity, water potential

## Abstract

Manure-derived organic amendments are a cost-effective tool that provide many potential benefits to plant and soil health including fertility, water retention, and disease suppression. A greenhouse experiment was conducted to evaluate how dairy manure compost (DMC), dairy manure compost-derived vermicompost (VC), and dehydrated poultry manure pellets (PP) impact the tripartite relationship among plant growth, soil physiochemical properties, and microbial community composition. Of tomato plants with manure-derived fertilizers amendments, only VC led to vigorous growth through the duration of the experiment, whereas DMC had mixed impacts on plant growth and PP was detrimental. Organic amendments increased soil porosity and soil water holding capacity, but delayed plant maturation and decreased plant biomass. Composition of bacterial communities were affected more by organic amendment than fungal communities in all microhabitats. Composition of communities outside roots (bulk soil, rhizosphere, rhizoplane) contrasted those within roots (endosphere). Distinct microbial communities were detected for each treatment, with an abundance of *Massilia*, *Chryseolinea*, *Scedosporium*, and *Acinetobacter* distinguishing the control, vermicompost, dairy manure compost, and dehydrated poultry manure pellet treatments, respectively. This study suggests that plant growth is affected by the application of organic amendments not only because of the soil microbial communities introduced, but also due to a synergistic effect on the physical soil environment. Furthermore, there is a strong interaction between root growth and the spatial heterogeneity of soil and root-associated microbial communities.

## 1. Introduction

Reducing the volume of organic wastes that end up in landfills is a critical challenge in the battle to mitigate climate change [1]. Using manure and other agricultural biproducts as a fertilizer or soil conditioner provides a sustainable solution for reducing waste and minimizing emissions while improving soils and recycling nutrients for plant growth. Over the past two decades, use of manure-derived fertilizer has increased on vegetable farms, much of which can be attributed to an increase in composted manure application [2].

Growers utilize manure-derived fertilizers and other organic amendments as a cost-effective tool to provide crop nutrition [3], stimulate microbial activity [4], manage plant pathogens [5], and improve soil physical and hydrological properties [6]. Despite their cost-effectiveness and availability, raw manures are generally avoided because they may emit volatile organic acids that are phytotoxic [5].

Compost is the product of a controlled aerobic process that degrades organic waste to stable material [7] with the resident microbial community mediating the biodegradation and conversion processes [8]. The USDA National Organic Program requires that compost piles maintain a thermophilic phase (maintain temperatures between 55 and 77 °C) for a minimum of 15 days and are turned a minimum of five times to ensure lethal conditions for resident pathogens [9] in windrow-produced compost. Alternatively, vermicomposting is a non-thermophilic composting process that involves joint action by earthworms and microorganisms [10]. Given the absence of a thermophilic stage, vermicomposting alone does not meet organic certification standards for processing manure. Compost-derived vermicompost, however, overcomes this limitation by combining the thermophilic phase of traditional windrow composting (thus meeting pathogen reduction standards) with a secondary vermicomposting curing phase. For brevity, we refer to this dairy manure compost-derived vermicompost simply as “vermicompost”.

Organic amendment is a broad term that includes manure-derived fertilizers that are not composted but instead heat-treated to eliminate pathogens and allow for application during the crop growing season [11]. This process is not synonymous with the successional phases and biological reactions that define composting [7], and the resulting product demonstrates very different physical, chemical, and biological properties [8]. For example, dehydrated poultry manure pellets, derived from a mix of manure, feathers, and bedding, are ground, heat-treated, and pelletized for commercial use. However, non-composted poultry manure-derived products can contain pathogens, heavy metals, antibiotics, and antibiotic-resistant genes that pose a threat to plant, human, and environmental safety [12].

There are two mechanisms by which compost can affect a soil microbiome: by modifying the abiotic soil environment and by adding microbes present in the compost to the soil. Application of organic amendments to soil can induce very different responses that are dependent on properties of the materials themselves. Organic amendments modify physical properties of the soil to which they are applied by decreasing bulk density and increasing soil organic matter, soil porosity, water infiltration, and water holding capacity [13]. These factors impact the amount of water a soil can hold in soil pore spaces. Both too little or too much water in soil pore spaces can be detrimental, leading to either drought stress and/or anaerobic conditions, respectively [14]. Changes to soil pore size distribution resulting from the application of these materials [15] directly impact habitat availability for different types of soil microorganisms. Among the most influential factors in shaping soil microbial community composition are soil water content [16] and the inherent physiochemical properties of organic amendments [17], which ultimately modify soil structure and hydrologic dynamics.

The microbial community composition of composts is determined by differences in feedstock (i.e., carbon and nitrogen sources), method (i.e., windrow, aerated static pile, vermicompost), and duration (i.e., successional stage, maturation) [8]. Each of these factors shape microbial communities by favoring organisms with varying affinities for different substrates or environmental conditions [5]. The microbial community of the finished compost, in turn, significantly influences the microbial community of the soil to which it is applied [18]. Microbial communities present in these materials possess a variety of tactics to compete with and defend against the resident soil microbial community for habitat and resources once incorporated into soil. The inoculation or stimulation of certain bacteria or fungi with known benefits to soil or plant health, referred to as beneficial microorganisms (BMs) or plant growth-promoting rhizobacteria (PGPR), are significant potential benefits of organic amendment application. The ability of BMs to compete with pathogens directly, via parasitism, antagonism, or antibiotic production, or indirectly, by inducing systemic defense responses in plants, is well documented (e.g., [19,20]. BMs and pathogens compete for habitat and resources in the areas immediately surrounding, on the surface of, or inside the tissue of plant roots [21].

While microorganisms compete for habitat and resources, plants themselves are not idle observers. The area immediately surrounding the plant root is inhabited by a unique population of microorganisms that are attracted to chemicals released from plant roots. Plants have developed the ability to modify the chemical composition in root exudates to favor organisms with affinities for certain compounds [22]. Microorganisms rapidly utilize these compounds as they are secreted from roots, fostering a highly active microbial community in the area immediately surrounding the root. Plants expend a considerable amount (up to 40%) of their photosynthesis-derived carbon to foster interactions with microorganisms in the rhizosphere [22]. In return, these microorganisms increase the availability and uptake of plant nutrients [23,24], induce changes in root growth [25], enhance plant tolerance to abiotic stress [24,26], and promote plant growth by synthesizing and excreting phytohormone analogs [27,28].

Communities of soil microbes are modified by the presence of plant roots and can be divided into four compartments or microhabitats: bulk soil, rhizosphere, rhizoplane, and endo(rhizo)sphere [29]. The factors responsible for shaping microbial community composition differ among each of these microhabitats. Bulk soil contains a reservoir of microorganisms with a community shaped primarily by soil type, vegetation history, and environmental factors [29]. In the rhizosphere, roots deposit carbon and organic acids, thus adding microbial food and reducing pH, and deplete the immediate environment of moisture, oxygen, and nutrients [29]. Rhizoplane microorganisms are selected from the rhizosphere largely based upon their ability to compete for a limited habitat and resources on the root surface [20,21,29]. Finally, community composition in the endosphere is also largely based upon abilities and characteristics of the organisms themselves. These organisms display the greatest level of specialization, having developed the ability to physically invade and inhabit root tissue, although their selection and enrichment is limited to the organisms present in other pools [29,30].

We know that certain manure-based fertilizers exhibit plant growth promoting properties, but we do not fully understand the complex interactions among soil physical properties, microbes, and plants that occur after their amendment and how these interactions shape agricultural outcomes. Application of organic amendments changes community composition at nearly all levels of taxonomic rank, from phylum to subspecies [31,32,33,34,35]. Nonetheless, differences in soil types, crop species, and properties of organic amendments make it difficult to compare results among studies.

The study was designed to test the hypothesis that manure-based vermicompost promotes plant growth at least as well as mineral fertilizer and is superior to windrow-based dairy manure compost or heat-treated poultry pellets. We conducted a greenhouse experiment to grow tomato (*Solanum lycopersicum*) with three organic amendment treatments (dairy manure compost, dairy manure-derived vermicompost, dehydrated poultry manure pellets) and a conventionally fertilized control. We measured plant growth and soil physical properties, and characterized the bacterial and fungal communities of the compartmentalized root microbiome using high-throughput amplicon sequencing of 16S rRNA and ITS-1 genes, respectively.

## 2. Materials and Methods

### 2.1. Experimental Design

We chose tomato because it is a popular cash crop grown both in field and hoophouses (for season extension). Furthermore, it allowed comparison to prior studies that focused on growth promotion by vermicompost [36]. Seeds of tomato (*Solanum lycopersicum*) varietal “Mountain Fresh Plus F1” (Johnny’s Selected Seeds, Winslow, ME, USA) were planted into separate 72 cell trays with a growing mixture amended as one of four treatments: dairy manure compost (DMC), dairy manure-derived vermicompost (VC), dehydrated poultry manure pellets (PP), and an untreated (conventionally fertilized) control (UC). After 30 days, 10–15 cm tall seedlings were transplanted into C100 (0.4 L), C200 (0.9 L), and C300S (1.6 L) Elite Custom blow-molded nursery pots (Nursery Supplies, Chambersburg, PA, USA) filled with field soil amended as one of the four treatments: DMC, VC, PP, and UC. Plants were grown in containers of three incrementally larger volumes to allow unrestricted root growth in those grown for one, two, or three weeks after transplant. Single plants were arranged as experimental units in blocks by harvest time in a completely randomized design. The entire experiment was repeated twice. Ten replicate plants were harvested from each treatment at each of four times (0, 7, 14 and 21 days after transplant) for a total of 160 experimental units per experimental replication. Of the 10 replicate plants destructively harvested per treatment-time combination, five were assessed for morphology and three sampled for characterization of the root microbiome with two extra replicates available as backups in the event of error. For each plant root system, multiple subsamples were collected from each of four microhabitats: bulk soil, rhizosphere, rhizoplane, and endosphere.

### 2.2. Growth Conditions

Seedlings were grown in a greenhouse at 21.7 °C day and 17.8 °C night temperatures with a range of 2.2 °C. Root-zone bench heating was provided at 26.7 °C to increase the probability of germination and encourage root growth. Mature seedlings were transplanted and transferred to an adjacent greenhouse module at 23.9 °C day and 18.3 °C night temperatures with a range of 1.65 °C. High-pressure sodium lamps provided supplemental lighting to maintain a photoperiod of 16 h from dawn throughout the experiment. Containers were spaced at appropriate distances to prevent crowding or shading between plants.

Containers with field soil were irrigated by hand two to three times per week with greenhouse tap water of pH 7 and electrical conductivity of 0.2 μS/cm. Soil volumetric water content (VWC) was used to dictate irrigation practices with irrigation events targeted for the VWC value observed at a matric potential of −300 kPa in each soil. At the time of each watering event, VWC was measured by total domain refractometry (TDR) using a ThetaProbe ML3 Soil Moisture Sensor and HH2 Soil Moisture Reader (Delta-T Devices, Cambridge, UK).

### 2.3. Organic Amendments

The treatments chosen are popularly used amendments in field vegetable production in the northeastern U.S. Vermicompost and dairy compost were obtained from Worm Power (Avon, NY). Worm Power compost feedstock is sourced from a local cow dairy that feeds a nutritionally consistent ration and provided sawdust bedding. Worm Power product was used because it is the same source as that used in earlier research [36,37]. Dairy manure with a small amount of silage is composted in an aerated static pile for an average of 40 days before the material is layered on top of a continuous flow vermiculture bed. The bed is densely populated with composting worms (*Eisenia fetida*) that digest the material for approximately six weeks before it is harvested as finished vermicompost. Both dairy manure-based products are produced with an identical recipe that is consistent throughout the year. These treatments were applied at the rate of 20% volume per volume of soil, as suggested by prior research [36,38]. Dehydrated, granulated 3-2-3 poultry manure purchased from Espoma (Millville, NJ, USA) was mixed 10% by volume with vermiculite to increase aeration before adding 20% volume of the mixture to soil. Control treatments were supplemented with 15-9-12 Smart-Release Plant Food Plus Outdoor & Indoor (Scotts Miracle-Gro Company, Marysville, OH, USA) at 8.75 g per liter of soil as suggested by the manufacturer.

### 2.4. Growing Media

Fort Vee growing mix (Vermont Compost Company, Montpelier, VT, USA) served as the base medium for germination and seedling growth. The growing mix was amended in a volumetric ratio unique to treatment (Table 1). These materials were standardized for N content based on volume as a horticulturist would use materials, but which translates into unequal levels based on weight (Table 1).

Field soil was used in the containers for the transplants. Approximately 0.75 cubic meters of field soil was collected from a local agricultural research site (44°26′39.0″ N, 73°11′23.9″ W). Soil was steam pasteurized in a 14MS Media Steamer Cart using a 210 Steam Aerator (Hummert International, Earth City, MO, USA) at 165 °C for four hours on two consecutive days to avoid the possibility of soil pathogens as a confounding factor. Steamed soil was rested for a few weeks to equilibrate in gas emission and microbial community before commencing with the experiment. This soil has been well characterized for related experiments (Neher, 2019). Indigenous microorganisms were extracted from the raw soil via soil extract and re-applied to the pasteurized soil after allowing the soil to cool for several days. Briefly, soil extract was prepared by diluting 250 g of soil in 2.5 L deionized water in a four liter Erlenmeyer flask. The flask was covered and placed in a C1 Orbital Platform Shaker (New Brunswick Scientific, Edison, NJ, USA) for 24 h. The shaken solution was vacuum filtered to 10 μm in a Buchner funnel. The filtered solution was diluted to 5 L and applied to the pasteurized soil in the steam cart once it had cooled. Lastly, the soil was sieved through a 2 × 2 cm screen to remove large organic and mineral debris and to homogenize the base soil mixture. Ten percent (*v*/*v*) vermiculite was added to the base soil mixture to provide structure and promote drainage (Table 2).

### 2.5. Soil Physical Properties

Pore size distribution of the soil was determined with a water release curve for each treatment mixed with soil. Samples were gently packed into 5 cm diameter × 7.5 cm tall cylinders on a porous ceramic pressure plate (Soil Moisture Equipment Corp, Santa Barbara, CA, USA). Soils were saturated for 24 h before pressure was applied at an amount equivalent to soil matric potentials of −40, −100, −200, −300, and −500 kPa. These matric potentials are representative of field capacity (−30 kPa) and gradually decreasing water availability to water deficit stress in common crops (−200 to −500 kPa). A single 1 cm diameter × 7 cm tall soil core was taken from each of three replicate cylinders per treatment after 48 h at a specific matric potential. These soil cores were weighed fresh and after drying at 90 °C for 72 h as a measure of gravimetric water content at each matric potential. Water released at a range of matric potentials provided a calibration curve for the time domain reflectometry (TDR) probe (Figure S1).

### 2.6. Plant Growth

Plant growth was measured as total plant biomass (shoots and roots) and root length density (RLD) was measured as a proxy for the probability of roots intercepting microbes originating from compost mixed into soil. Each plant was severed at the hypocotyl upon harvest. The belowground root mass was carefully separated from the soil mixture in a 20 L bucket of tap water by gently massaging the soil away from the roots. The washed root mass was cleaned with a fine mist of water to remove any debris. Both the aboveground and belowground biomass were dried at 60 °C for 96 h and summed for expression as grams of total dry mass per plant. Root length density (RLD) per plant was measured by placing roots in 250 mL distilled water within a 22 × 28 cm plexiglass tray on an Epson Perfection V370 scanner (Seiko Epson Corporation, Suwa, Nagano, Japan). A high-resolution digital image of the sample was manipulated using AxioVision SE64 software (Zeiss Group International, Oberkochen, Germany) to express RLD as length of root per volume of soil (cm root length per cm^3^ soil).

### 2.7. Root Microbiome

Roots of turgid plants were harvested consistently from soil that had not been watered for at least 4 h. Belowground samples were subdivided along a spatial gradient for comparison of microorganisms residing in four microhabitats: bulk soil, rhizosphere, rhizoplane, and endosphere [29]. The root mass for each plant was removed from its container over a sterile metal collection basin to prevent cross contamination. Bulk soil that freely dislodged from the root mass during removal from the container was collected, from which three 0.04 g subsamples were taken. The root mass was gently shaken over a second basin to dislodge most soil from the roots. The rhizosphere soil that remained intact with the root was sampled in triplicate using a sterile cotton swab dipped in twice-autoclaved Milli-Q (Millipore Sigma, Burlington, MA, USA) filtered water. Each cotton swab covered with rhizosphere soil was cut from the swab spindle with flame-sterilized scissors and placed into 2 mL polypropylene microcentrifuge tubes with caps (Fisher Scientific International, Hampton, NH, USA) that were briefly vortexed and sonicated for 20 min in a VWR Model 150D sonicator bath (VWR International, West Chester, PA, USA). The cotton swab was removed and the suspension with soil was kept as the representative subsample of the rhizosphere community. Finally, five 2 cm long growing root tips were excised from each plant using flame-sterilized scissors. Excised roots from each plant were pooled, rinsed in twice-autoclaved Milli-Q water, and sonicated for 20 min. The post-sonication suspension was kept as the representative subsample of the rhizoplane community removed from the root (endosphere). With the exception of endosphere samples, each of three subsamples per microhabitat (bulk soil, rhizosphere, rhizoplane) per plant were transferred, in a dark room, to a sterile 2 mL polypropylene microcentrifuge tube filled with 1.4 mL sterile-filtered phosphate buffer saline containing 3.5 μL of 40 μM propidium monoazide (PMA) dye solution (Biotium, Inc., Fremont, CA, USA). Samples were prepared in triplicate with small volumes of soil and solution to avoid adsorption of PMA to soil particles and allow the solution to mix thoroughly during light exposure, ensuring a complete photolytic reaction of the PMA [39,40]. Endosphere samples were removed from the solution with flame-sterilized forceps and transferred to QIAGEN PowerBead Tubes from a DNeasy PowerSoil Kit (QIAGEN, Germantown, MD, USA), and immediately stored at −80 °C.

### 2.8. PMA Photolysis and DNA Extraction

PMA photolysis and DNA extraction methods followed protocols described by Carini et al. [39] and Lauber et al. [41], respectively. Briefly, a 600 W halogen lamp was placed 20 cm above an ice bath secured on a C1 Orbital Platform Shaker (New Brunswick Scientific, Edison, NJ, USA). Tubes containing bulk soil and rhizosphere samples were placed on ice, shaken, and illuminated 30 s on/30 s off four consecutive times. Shaking ensured that the contents of the tubes experienced even light exposure. The ice bath served to secure the tubes, keep the solutions cool from the heat of the lamp, and provide reflection of light. After incubation, tubes were stored at −80 °C until DNA was extracted.

DNA was extracted from bulk soil, rhizosphere, rhizoplane, and endosphere samples using a DNeasy PowerSoil Kit (QIAGEN, Germantown, MD, USA). Specifically, 850 μL from each 2 mL sample tube was transferred to PowerBead Tubes, placed in a 65 °C water bath for 10 min, and then shaken horizontally for 2 min at maximum speed with the MoBio vortex adapter. A quantity of 500 μL of the bead-beaten solution was transferred, undergoing subsequent steps to isolate and purify DNA as directed by the manufacturer. Triplicate subsamples of bulk soil (per plant), rhizosphere (per plant), rhizoplane (per treatment–time combination), and endosphere (per treatment–time combination) were extracted individually. The resulting purified DNA was pooled for each microhabitat to obtain a sufficient concentration of DNA for sequencing. Pooled 300 μL purified DNA samples were shipped overnight express to the University of Colorado at Boulder for amplicon sequencing.

### 2.9. Amplicon Sequencing

Samples were amplified using 515F/806R primers targeted for the V4 region of the 16S rRNA gene for bacteria and archaea, and ITS-1/ITS-2 primers to amplify the ITS-1 gene for fungi. Samples were amplified in triplicate and adjusted to equimolar concentrations. One microliter of genomic DNA was added to 13 µL of PCR-grade water, 10 µL of Prime Hot Master Mix, 0.5 µL of reverse primers, and 0.5 µL of forward primers. PCR was carried out in 35 thermocycles of 94 °C for 45 s, 50 °C for 60 s, and 72 °C for 90 s.

Quality filtering and clustering of sequences into exact sequence variants (ESVs) was performed using the DADA2 pipeline [42]. The DADA2 pipeline contains a pre-processing step which demultiplexes with the *idemp* program, removes sequences with ambiguous bases, and removes any primers with *cutadapt* [43]. Data were then filtered and trimmed for quality, sequence variants inferred, chimeras removed, singletons removed, and taxonomy assigned using the Greengenes v13.8 database for bacterial and archaeal 16S rRNA gene sequences [44] or the UNITE v7.2 fungal ITS database for fungi [45]. Mitochondrial, chloroplast, and eukaryote sequences were removed from 16S data. ESVs not assigned to kingdom or phylum were removed prior to downstream analyses. The 16S data were rarefied to 11,441 sequences per sample, trimming the data from 187 to 174 samples. The ITS data were rarefied to 1028 counts, trimming the data from 185 to 168 samples. The final 16S and ITS analyses included 18,038 and 887 ESVs, respectively.

### 2.10. Statistical Analysis

Plant growth data were analyzed as a full model two-way analysis of variance (ANOVA) with plant phenology (sampling week) and treatment as independent variables. A linear regression was performed to quantify the association between volumetric water content (measured by TDR) and water potential. Plant available water was analyzed as a one-way ANOVA with treatment as an independent variable followed by a Tukey post-hoc means comparison. The ANOVAs and linear regression were performed using GraphPad Prism Ver. 9.1.2. (https://www.graphpad.com/, accessed on 7 July 2021).

The *mctoolsr* package in RStudio Version 1.1.463 (https://github.com/leffj/mctoolsr, accessed on 1 June 2021) was used to create plots of richness (number of unique ESVs per sample) and biplots of principal coordinate analyses (PCoA) using Bray–Curtis dissimilarity matrices. Permutational multivariate analysis of variance performed in R provided pseudo-F- and *p*-values from Monte Carlo permutation tests between treatments and root microbiomes with harvest time as a block. Post hoc comparisons using Kruskal–Wallis tests between factor pairs provided R^2^ and false discovery rate (FDR) corrected *p*-values using the *adonis* function in the R package *vegan*.

## 3. Results

### 3.1. Plant Growth

**Figure 1:**
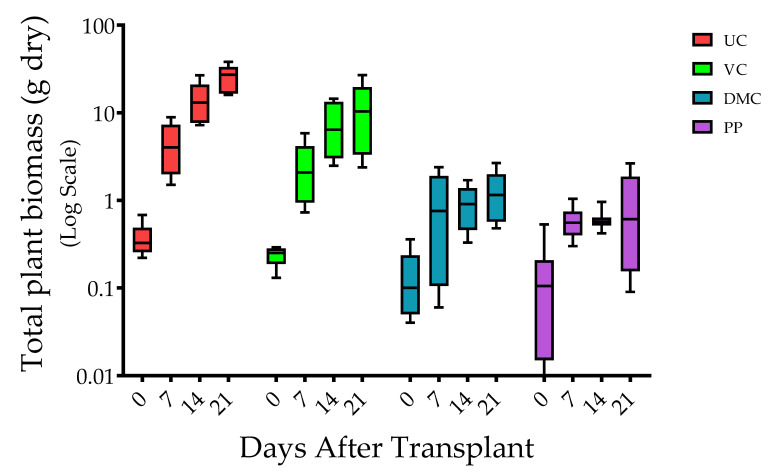
Box and whisker plot of total dry biomass of plants at 0, 7, 14, and 21 days post-transplant (*n* = 10). The box represents 25th, 50th (median), and 75th percentiles, and the whiskers represent minimum to maximum values. Treatments represent growing mix and soil with amendment of vermicompost (VC, 20% *v*/*v*), dairy manure compost (DMC, 20% *v*/*v*), or dehydrated poultry manure pellets (PP, 10% *v*/*v*) compared to an untreated (conventional fertilizer) control (UC).

Dry total plant biomass varied among harvest times (*p* ≤ 0.0001), treatments (*p* ≤ 0.0001), and the interaction of harvest time and treatment (*p* ≤ 0.0001) (Figure 1). Plants of the UC treatment had the greatest total plant biomass at each harvest time. Total plant biomass decreased progressively with amendment of VC, DMC, and PP. The magnitude of differences among treatments increased through time. The pattern was similar whether expressed as plant height or total biomass.

**Figure 2:**
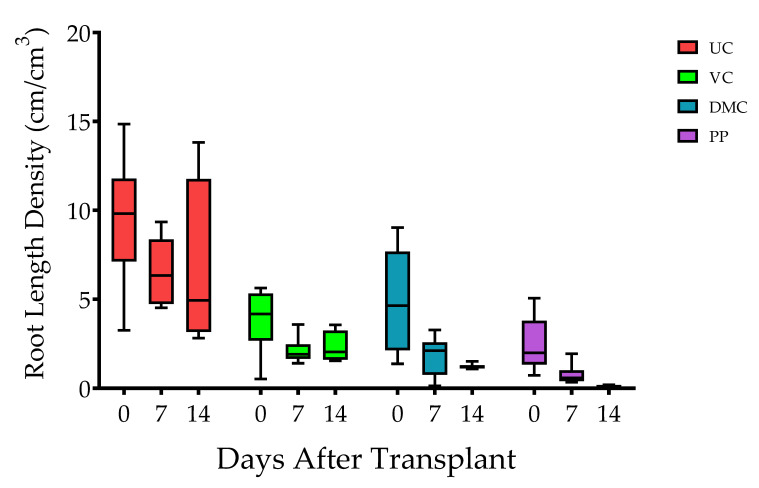
Box and whisker plot of root length density through time (*n* = 5). The box represents 25th, 50th (median), and 75th percentiles, and the whiskers represent minimum to maximum values. Error bars represent standard error of the mean. Treatments represent growing mix and soil with amendment of vermicompost (VC, 20% *v*/*v*), dairy manure compost (DMC, 20% *v*/*v*), or dehydrated poultry manure pellets (PP, 10% *v*/*v*) in comparison to an untreated (conventional fertilizer) control (UC).

Root length density (RLD) differed among harvest times (*p* ≤ 0.0001) and treatments (*p* ≤ 0.0001) but not the interaction of time and treatment (*p* = 0.8752) (Figure 2). Plants of the UC treatment had the greatest RLD at each harvest time. RLD of seedlings decreased progressively with amendment of DMC, VC, and PP. RLD decreased among all treatments after transplant. RLD decreased progressively with increasing days after transplant among the plants in the DMC and PP treatments (Figure 2).

### 3.2. Soil Physical and Hydrological Properties

**Figure 3:**
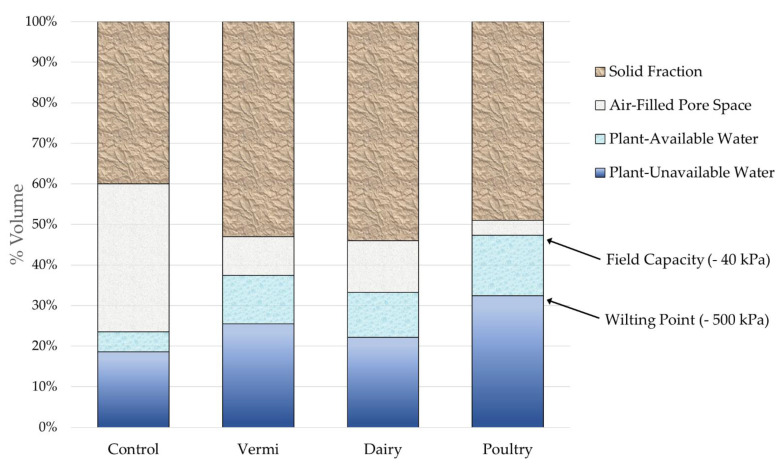
Solid, air, and water fractions of soils with or without organic amendments as a percentage of total volume. Values represent means (*n* = 3). Total volume of core = 147.44 cm^3^. Treatments represent soil amended with vermicompost (VC, 20% *v*/*v*), dairy manure compost (DMC, 20% *v*/*v*), or dehydrated poultry manure pellets (PP, 10% *v*/*v*) compared to an untreated (conventional fertilizer) control (UC). Field capacity is the amount of water content held in soil after excess water has been drained by gravity. Wilting point represents soil water that is no longer extractable by plants because it is held too tightly to soil particles by capillary action.

Water release at defined matric potentials was associated linearly with TDR probe measurements (Figure S1). Although TDR probe measurements were precise, they consistently underestimated gravimetric water content by approximately 11%. Plant available water content was least in the UC, intermediate in DMC and VC, and greatest in PP treatments (*p* ≤ 0.0001). UC soil had the least total pore space, total water content, and plant available (between −40 and −500 kPa) water content (Figure 3). Soils treated with organic amendments were more porous than the UC and held more plant available and unavailable water content. Soil amended with VC had similarly greater water holding capacity as soil amended with DMC (*p* = 0.5544). Soil amended with PP contained relatively little air (8%) compared to water (92%) in pore spaces.

### 3.3. 16S Community Composition

16S community composition differed among amendments (pseudo-F = 19.9, *p* = 0.001) and microhabitats (pseudo-F = 34.1, P_FDR_ = 0.001), and amendment–microhabitat combinations (pseudo-F = 1.9, P_FDR_ = 0.001) (Figure S3). All pairwise comparisons between amendments were significantly different from each other (P_FDR_ = 0.002). All but one of the microhabitats differed from one another (P_FDR_ ≤ 0.01). Specifically, the rhizoplane and endosphere communities were indistinguishable (P_FDR_ = 0.529).

**Figure 4:**
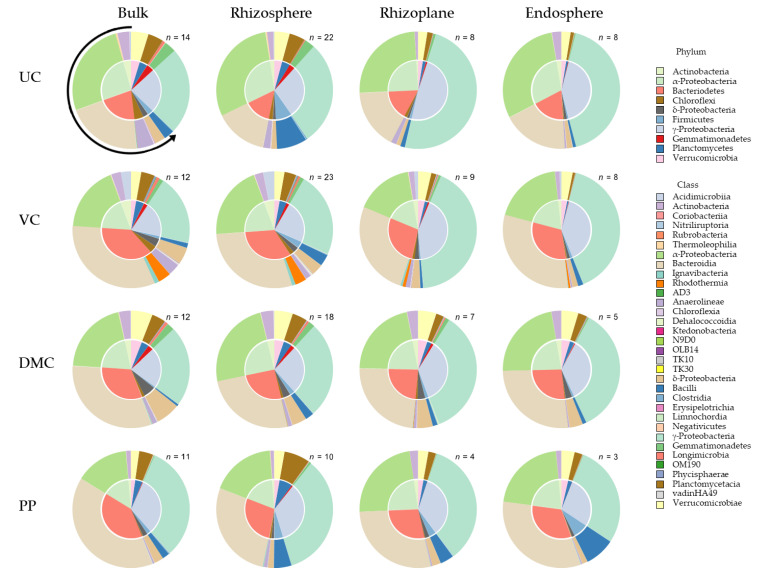
Community assembly of bacteria and archaea by 16S amplicon sequencing for microhabitat (columns) for each of four treatments (rows). Microhabitats are illustrated by column: bulk soil, rhizosphere, rhizoplane, and endosphere. Treatments represent growing mix and soil with amendment of vermicompost (VC, 20% *v*/*v*), dairy manure compost (DMC, 20% *v*/*v*), or dehydrated poultry manure pellets (PP, 10% *v*/*v*) compared to an untreated (conventional fertilizer) control (UC). 16S community composition is illustrated by taxonomic phyla (inner pie) and class (outer pie). Data presented represent pooled samples from all harvest times (*n* = 174). The arrow around the upper left pie indicates directionality of the legend (counterclockwise) and where the slices start (12 o’clock).

Generally, the 16S community became less diverse with closer proximity to the root. There were distinct differences in community composition among treatment and, furthermore, root microhabitat within each growing medium (Figure 4). Soils amended with VC had the most unique bacterial community. Total variation among treatments was slightly greater in soil than it was for the growing mix (“control”). Bacterial community composition differed most between growing mediums for the UC treatment and least for the PP treatment.

Bacterial community composition varied among microhabitats within each treatment (Figure 4). Root microhabitats within each treatment segregated into pairs: the bulk soil and rhizosphere communities, and the rhizoplane and endosphere communities. This pairing was distinct in all but one treatment, in which the rhizoplane and endosphere communities differed in soils amended with DMC. The rhizoplane and endosphere bacterial communities in amended soils varied more from those of the UC soil than did the bulk soil and rhizosphere bacterial communities.

Bacterial communities were dominated by the phyla Bacteroidetes and Proteobacteria. Proteobacteria were primarily from α- and γ-Proteobacteria classes (Table S1). The percentage of δ-Proteobacteria was more similar to that of the other major phyla present: Acidobacteria, Actinobacteria, Chloroflexi, Firmicutes, Gemmatimonadetes, Planctomycetes, and Verrucomicrobia.

The UC treatment hosted the least abundance of Bacteroidetes and the greatest abundance of Proteobacteria (Figure 4). Organic amendments increased the abundance of Bacteroidetes. PP and VC amendment led to the first and second greatest abundance of Bacteroidetes, respectively. The third most abundant phylum explained 4% to 5% of the bacterial community in each treatment. The third most abundant phylum in the UC, VC, and DMC, and PP treatment was Firmicutes, Actinobacteria, Verrucomicrobia, and Planctomycetes, respectively (Figure 4).

Treatment differences were distinct at finer taxonomic resolution. Seven of the ten most abundant taxa were γ-Proteobacteria: *Acinetobacter*, *Luteimonas*, *Rheinheimera*, *Pseudomonas*, *Steroidobacter*, and two ESVs from the genus *Massilia* (Table 3) Two ESVs identified as *Chryseolinea* were the only representatives of the Bacteroidetes phylum. *Devosia*, the only representative of α-Proteobacteria, was the most ubiquitous organism, present in 155 of 174 samples and amongst the 10 most abundant taxa in all treatments but the PP. Only *Pseudomonas* was amongst the 10 most abundant genera in all four treatments (Table 3).

The abundance of specific genera provided the greatest distinction among treatments. The most abundant genus in the UC treatment was *Massilia*. This genus accounted for less than 0.5% of the bacterial community in other treatments. The VC treatment hosted the greatest abundance of *Rheinheimera* and more than ten times the abundance of *Chryseolinea* of any other treatment. The DMC treatment hosted the greatest abundance of *Asticcacaulis*, *Devosia*, *Luteimonas*, and more than three times the abundance of *Steroidobacter* of any other treatment. The PP treatment had more than eight times the abundance of *Acinetobacter* of any other treatment. Amending with PP also led to the greatest abundance of Bacteroidetes genera of the orders Sphingobacteriales (*Arcticibacter*, *Pedobacter*, *Sphingobacterium*) and Flavobacteriales (*Flavobacterium*, *Fluviicola*) (Table S1).

#### 3.3.1. Plant Phenology

Sampling each treatment before germination provided a baseline microbial community that was influenced by the presence of an actively growing seedling. The unamended growing mix was dominated by an unidentified genus from the Chitinophagaceae family. The abundance of this organism decreased in each of the amended treatments. The growing mix amended with VC hosted a greater abundance of *Tumebacillus* and *Chryseolinea*, whereas the growing mix amended with PP hosted a greater abundance of *Acinetobacter* and *Streptomyces*. Thirty days after germination, the growing mixes amended with VC, DMC, and PP were distinguished by an abundance of *Chryseolinea*, *Steroidobacter*, and *Roseimaritima*, respectively.

Differences among treatments were even greater after transplant (Figure 4). Despite relatively little abundance in the growing mix, the UC treatment hosted the greatest abundance of *Massilia* after transplant to soil. *Chryseolinea* abundance continued to distinguish the VC treatment. Soil amended with DMC hosted the greatest abundance of *Asticcacaulis* and *Devosia*, whereas soil amended with PP was dominated by *Acinetobacter*.

#### 3.3.2. Root Microhabitat

The number of unique ESVs (taxonomic richness) was greatest in bulk soil and least in the root endosphere (Figure S2). The greatest variation in richness among organic amendments was in the rhizosphere. The proportion of the dominant phyla varied little among root microhabitat (Figure 4). Notably, γ-Proteobacteria were enriched with closer proximity to the root, whereas observation of this trend among other phyla was better depicted at finer resolution. The abundance of Proteobacteria, including *Cellvibrio*, *Devosia*, *Pseudomonas*, *Rheinheimera*, *Shinella*, and *Stenotrophomonas*, increased with closer proximity to the root (Table S2).

In the UC treatment, the abundance of *Massilia* in the rhizoplane and the endosphere was approximately four times that of the bulk and rhizosphere. In the DMC treatment, *Pseudomonas* and *Stenotrophomonas* were ten times and one hundred times more abundant, respectively, in the endosphere than bulk soil. Similarly, in the PP treatment, the abundance of *Shinella* and *Stenotrophomonas* increased linearly with closer proximity to the root. In contrast, several genera in the Bacteroidetes were more abundant in bulk and rhizosphere soil than they were inside roots or on the root surface. In the VC treatment, for example, *Chryseolinea* was more than twice as abundant in bulk and rhizosphere soils as it was on the rhizoplane.

### 3.4. Fungal Community Composition

**Figure 5:**
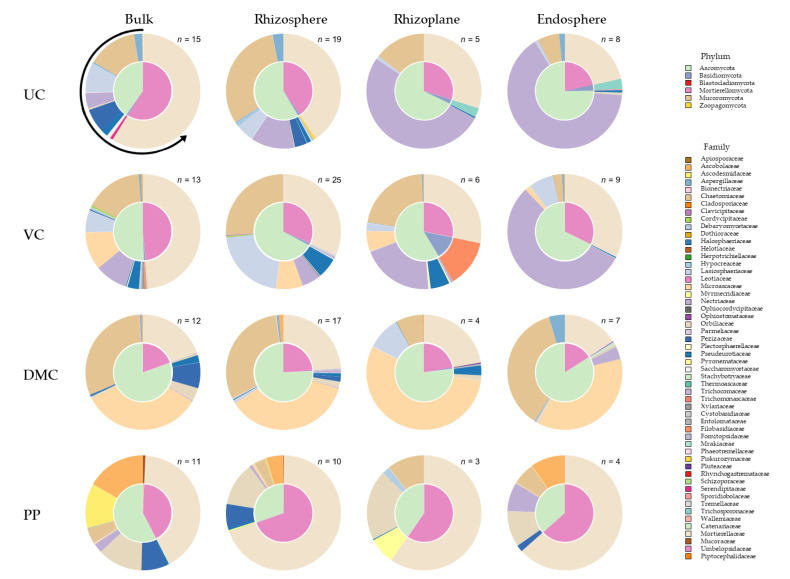
Community assembly of fungi by ITS amplicon sequencing for microhabitats (columns) for each of four treatments (rows). Microhabitats are illustrated by column: bulk soil, rhizosphere, rhizoplane, and endosphere. Treatments represent growing mix and soil with amendment of vermicompost (VC, 20% *v*/*v*), dairy manure compost (DMC, 20% *v*/*v*), or dehydrated poultry manure pellets (PP, 10% *v*/*v*) compared to an untreated (conventional fertilizer) control (UC). Fungal community composition is illustrated by taxonomic phyla (inner pie) and family (outer pie). Data presented represent pooled samples from all harvest times (*n* = 168). The arrow around the upper left pie indicates directionality of the legend (counterclockwise) and where the slices start (12 o’clock).

The fungal community composition of the VC and DMC treatments were the least and most different from the UC, respectively (Figure 5, Figure S3). Regardless of treatment, fungal communities varied between endosphere and bulk soil or rhizosphere (P_FDR_ = 0.02), but were similar among other pairs of microhabitats (P_FDR_ > 0.1). The treatments that had the healthiest plants (UC, VC) went from most diverse in the bulk soil to being dominated by the same family (Nectriaceae) in the endosphere. Nectriaceae contains the genus *Fusarium*. The microbial communities were quite different in the DMC and PP treatments.

Fungal communities were dominated by Ascomycota, Mortierellomycota, and Basidiomycota (Table 4). In addition, there were small populations (<1%) of Blastocladiomycota, Chytridiomycota, Mucoromycota, Rozellomycota, and Zoopagomycota. Amendments increased the abundance of Basidiomycota, especially DMC and VC (Table S3). The VC treatment also hosted a small population of Rozellomycota that was not found in any other treatments. DMC was the only amendment that increased Ascomycota abundance. PP was the only amendment to increase Mortierellomycota abundance, doing so considerably.

*Mortierella* was the most abundant genus in the UC and PP treatments (Table S3). The second most abundant genus in the UC treatment was *Fusarium*. This genus was moderately abundant in soils amended with VC. The third most abundance genus in the UC treatment, *Zopfiella*, increased with the amendment of DMC. Amending with DMC introduced *Scedosporium*, a genus absent from the UC treatment. Amending with PP increased the abundance of *Arthrobotrys*, found in little abundance in the other treatments, and *Cephaliophora*, which was not present in any other treatments.

The unamended growing mix was dominated by Mortierellomycota, specifically *Mortierella*. Amendments increased the abundance of Ascomycota. Amending growing mix with DMC increased Basidiomycota and introduced *Scedosporium*, a genus that was absent from other treatments. Seed germination increased the abundance of *Scedosporium*, which carried over into soil after transplant. Ascomycota was the most abundant phylum in all soils after transplant. Amending soil with VC and PP increased the abundance of Basidiomycota, whereas PP-amended soil hosted the greatest abundance of Mortierellomycota.

Manure-derived amendments increased richness from the untreated control (Figure S2). Richness generally declined as microhabitat was in closer proximity to the root (Figure S2). Microhabitats were generally less distinct among fungal ESVs than among bacterial ESVs (Figure 4 and Figure 5). In the UC treatment, *Mortierella* abundance was greatest in the bulk soil, whereas *Fusarium* was most abundant in closer proximity to the root (Table S4). *Fusarium* abundance followed a similar pattern in the VC treatment. Abundance of certain genera by microhabitat did not follow consistent patterns in soils amended with DMC or PP.

## 4. Discussion

Vermicompost promoted plant growth as much as the fertilizer controls. None of the composts received fertilizer inputs, suggesting vermicompost can obtain the results achieved by fertilizer, but without the fertilizer. This study demonstrates that vermicompost derived from dairy manure compost promotes plant growth more than traditional dairy manure compost and non-composted dehydrated poultry manure pellets. Bacterial and fungal communities in dairy manure compost-derived vermicompost are more mature and stable than those of dairy manure compost or poultry manure pellets [46,47] which correlates with less phytotoxicity [48]. This study contributes to the limited but developing knowledge of 16S and ITS communities associated with dehydrated poultry manure pellet and dairy manure-derived vermicompost amendment [31,33,36]. Poultry manure pellet amendment was inferior to both dairy manure-based products in this system. Despite its widespread use as a nitrogen-rich fertilizer, we found that poultry manure pellets dramatically altered the physical structure of soil to the extent that it impeded root growth. Furthermore, this study validates that the absence or insufficient duration of composting can result in a cascade of deleterious effects on crop and soil health [37,49].

The communities sequenced in this study were dominated by bacteria of the phyla Bacteroidetes and Proteobacteria. Other studies conducted on tomato plants report a community dominated by Proteobacteria [50,51,52,53,54] and Ascomycota, the dominant fungal phylum in this study [53]. In addition, Bacteroidetes have been observed previously in similar abundance in the tomato rhizosphere [51,54]. Several of these studies also documented the presence of Acidobacteria, Actinobacteria, Bacteroidetes, Chloroflexi, Gemmatimonadetes, Firmicutes, and Verrucomicrobia.

### 4.1. Soil Properties and Plant Growth

Contrary to the existing literature, amendment with manure-derived fertilizers was detrimental to plant growth in this study. Plants grown in soils without these amendments displayed more advanced maturation and greater height, biomass, and root length density than those treated with these amendments. Increasing water holding capacity of soils treated with manure-derived fertilizer amendments was correlated with less root growth and total plant biomass. Of those treated with manure-derived fertilizers amendments, only VC led to vigorous growth through the duration of the experiment, whereas DMC had mixed impacts on plant growth and PP was detrimental. Through time, the difference among treatments increased as the health of plants in soils amended with DMC and PP declined. Variability in the nutrient composition of these amendments may have also contributed to plant growth differences and should be quantified to provide additional support for these findings.

Soil amended with VC held less moisture than those amended with DMC or PP and demonstrated superior root growth as a result. VC is a finely divided peat-like material with excellent structure, porosity, aeration, and drainage [10]. Combining composting and vermicomposting reduces the electrical conductivity and C to N ratio of the material, thus reducing phytotoxicity and N immobilization, respectively [46]. The vermicompost process also increases nitrogen availability to plants by enhancing the nitrification of ammonium to nitrate [47]. As a result, VC amendment consistently demonstrates impacts on plant growth similar to those of fertilizer controls [38].

In contrast, plants grown in soils amended with DMC demonstrated symptoms indicative of compost immaturity. Phytotoxicity of immature composts can manifest symptoms including stunting, chlorosis, and limited root development [49]. Decomposition of labile compounds in immature composts consumes oxygen and immobilizes nitrogen [49,55]. Depletion of oxygen can inhibit root function [56] and microbial demand for nitrogen can stunt plant growth [57]. Immature composts also contain volatile organic acids and free ammonia, which inhibit seed germination and root growth [49]. The duration of the maturation phase of a compost is fundamental to reducing phytotoxicity [49].

Amendment with PP reduced drainage and resulted in anaerobic conditions that led to a cascade of deleterious effects on plant growth and soil microbes. Oversaturation and poor drainage limit air-filled pore space, causing injury and inhibiting function of roots by oxygen deficiency [56]. These roots were further damaged by herbivory from fungus gnat larvae, which were abundant only in soil amended with PP. Anaerobic microsites created by PP amendment can also lead to the reduction of nitrogen to nitrogenous gas [49]. Increases in nitrous oxide [58,59] and ammonia [60] have been observed after amendment with poultry manure-based amendments. Direct inhibition of root growth by volatilized ammonia has been observed shortly after the application of poultry manure [61].

### 4.2. Plant Phenology and Root Microhabitat

Composition of microbial communities varied by plant phenology and microhabitat. The influence of treatment on microbial communities was evident immediately after amendment. Composition shifted with seed germination and seedling growth, demonstrating the ability of certain introduced taxa, including *Scedosporium* in the DMC treatment, to colonize the rhizosphere and sustain growth through time. Changes in microbial communities occur upon root emergence [62] and throughout plant development [63]. Rhizosphere community composition also differed between growing mix, before transplant, and field soil, after transplant, an observation that was recently made in tomato [54]. After transplant, with the decline of the DMC and PP treatments, the impact of active root growth and exudation on the microbial community [22] was evident in the presence (UC, VC) or absence (DMC, PP) of a rhizosphere effect [29]. These findings align with the notion that plant health status imposes significant influence on the rhizosphere microbial community [64].

Partitioning by microhabitat in this study is similar to previous reports for tomato [51,52,53], e.g., relative abundance of γ-Proteobacteria genus *Pseudomonas* near the root [52]. *Pseudomonas* are often recognized as plant growth-promoting rhizobacteria (PGPR) with biocontrol potential [20,24,30,65]. However, closely related species of *Pseudomonas* are also prolific pathogens [66]. Limitations to the methods do not provide sufficient information to determine the functional role of *Pseudomonas* in this study. Other γ- Proteobacteria observed in this study that have PGPR potential include *Devosia* [67] and *Massilia* [35,68].

### 4.3. Treatment-Specific Microbial Communities

Unamended soil hosted fewer γ-Proteobacteria than α-Proteobacteria and fewer Bacteroidetes compared to soils treated with manure-derived fertilizer amendments. This finding coincides with the expected increase in Bacteroidetes abundance, and expected increase and decrease in γ-Proteobacteria and α-Proteobacteria abundance, respectively, in composts [8]. The control treatment was also distinguished by comparatively high abundances of the bacteria *Massilia* and the fungi *Mortierella* and *Fusarium*. *Massilia* may function to promote plant growth [68] but its abundance is inversely impacted by organic amendment application [69]. *Mortierella* abundance in bulk and rhizosphere soil is correlated with healthy roots [70] and is relatively abundant in poultry litter compost [33].

The abundance of *Fusarium*, a genus known for its predominant role as a plant pathogen [71], was unexpected due to the vigorous plant growth and larger root mass observed of the two treatments (UC and VC) in which its abundance was greatest. VC samples hosted little *Massilia* abundance but shared a greater abundance of *Mortierella* relative to the other treatments. Interestingly, both *Massilia* [35,72] and *Mortierella* [73] have been implicated in the suppression of *Fusarium* and other plant pathogens. *Massilia* abundance in the UC treatment was greatest in the rhizoplane and endosphere samples, suggesting this organism may suppress *Fusarium* by competing for habitat and resources.

Despite being known primarily as a plant pathogen, *Fusarium* also contains species that are non-pathogenic, or biocontrol agents against pathogenic Fusarium wilt of tomato [74]. Given the absence of disease symptoms and the correlation between *Fusarium* abundance and increased plant biomass, the *Fusarium* spp. observed in this study is related to the *F. fujikuroi* species complex, which includes pathogenic and non-pathogenic strains that survive saprophytically and promotes plant growth [75,76].

VC-amended samples had the greatest abundance of Bacteroidetes of each treatment. Bacteroidetes and Proteobacteria dominate vermicompost microbial communities [77] and are more abundant in dairy manure-derived VC than windrow or aerated static pile composting systems [8]. Passage of materials through the worm *Eisenia fetida* digestive tract selectively modifies bacterial communities [78], whereas secretion of mucus-covered fecal pellets increases soil aggregation and provides readily available carbon to increase soil microbial activity [79] and nutrient availability [47]. The genus distinguishing the VC treatment, *Chryseolinea*, is promoted by compost amendment [80] and seed-applied biostimulant application [81]. *Chryseolinea* demonstrates enrichment in tomato roots [82] and has also been implicated in pathogenic *Fusarium* suppression [83]. Organisms in the VC treatment that were abundant in the rhizoplane and endosphere, including *Pseudomonas* and *Rheinheimera*, have been isolated from vermicompost [84] and also demonstrate suppression against pathogenic *Fusarium* [85].

DMC amendment was distinguished by the abundance of organisms that may be associated with compost immaturity and manure feedstocks. These organisms include the bacteria *Asticcacaulis* and *Steroidobacter*, and fungi *Zopfiella* and *Scedosporium*. *Asticcacaulis* is a genus with an affinity for simple carbon sources [86] that are present in immature composts. *Scedosporium* is found abundantly during the early stages of mesophilic composting and absent in the mature product [87]. *Zopfiella* has been identified in compost recipes including manure [8] and *Steroidobacter* is among the most abundant genera in composts including dairy cow manure [88]. The abundance of these organisms supports the notion that this material was unsuitable for use as a soil amendment due to its immaturity.

Several genera favored in the PP treatment, including *Fluviicola*, *Flavobacterium*, and *Pedobacter*, are known to colonize soil amended with poultry manure-based amendments [33,34]. These bacteria of the orders Flavobacteriales and Sphingobacteriales, in addition to fungi of the phylum Mortierellomycota, are significantly more abundant in soils treated with poultry manure-based amendments than dairy manure-based amendments [34]. These organisms demonstrate copiotrophic lifestyles supported by the greater nitrogen content of poultry manure [34] and the labile nature of the non-composted PP product. The high nitrogen content of poultry manure-based amendments also supports the survival of human pathogenic bacteria in field environments [33], which can be transmitted to the consumer on fresh produce [89].

PP is a dehydrated product that is approved for organic production, which may give the impression that the product is physiochemically or biologically stable. However, our observations suggest that poultry manure pellets demonstrate properties similar to those of raw manure applied to soil. Some of the organisms associated with PP in this study and with poultry manure-based amendments in previous research [33,34] are also associated with nitrogen volatilization [90], which occurs after the application of poultry manure to soil [58,59,60,61].

The most abundant ESV in the PP treatment, *Acinetobacter*, also colonizes poultry manure [91] and tomatoes grown in soil amended with PP [31]. The ability of *Acinetobacter* to outcompete other organisms has been observed in the tomato phyllosphere [52] and on tomato roots after infection with the pathogen *Ralstonia solancearum* [92]. *Acinetobacter* also includes anaerobic denitrifying species [93], which would coincide with the overly saturated growing environment observed of this treatment.

## 5. Conclusions

This study suggests that plant growth is affected by application of organic amendments not only by the soil microbial communities introduced, but also due to a synergistic effect on the physical soil environment. Furthermore, there is a strong interaction between root growth and the spatial heterogeneity of soil and root-associated microbial communities. Greater use of composts made from manure converts organic waste to a useful resource that promotes vegetable production and reduces fossil fuel inputs. Compost is an important component of a sustainable agriculture, not only to close the nutrient loop, but also as a means to reduce the environmental footprint of animal-based agriculture [94].

The varied response to organic amendment application reflects the inherent variability among manure-derived fertilizers and the dramatic impact these differences have on plant growth, soil physical properties, and soil microbial community composition. Successful utilization of these products to improve soil health and crop production will require a concerted effort to more comprehensively characterize these materials and the microbial communities that result from their application to the soil environment. Although this study highlights the widespread need for this research, it also successfully demonstrates the value of vermicomposting to stabilize manure-derived fertilizer products. The greater adoption of vermicomposting as a compost curing phase would serve to provide growers with a reliable alternative to synthetic fertilizer for promoting plant and soil health.

## Figures and Tables

**Table 1:** Composition of growing medium used in plug trays for germination and seedling growth.

Treatment	% Volume Fort Vee	% Volume Organic Amendment	Adjusted N (g L^−1^)
Control	100	0	1.3
Vermicompost	80	20	7.5
Dairy manure compost	80	20	7.5
Poultry pellets	80	10 *	5.5

* 10% of commercial product in the soil with an additional 10% vermiculite.

**Table 2:** Composition of soil mixtures used in containers for transplants.

Treatment	% Volume Field Soil	% Volume Amendment	% Volume Vermiculite	Bulk Density (g/cm^3^)
Control	90	0	10	0.99
Vermicompost	70	20	10	0.88
Dairy manure compost	70	20	10	0.83
Poultry pellets	70	10	20 *	0.85

* 10% vermiculite added to increase drainage.

**Table 3:** Ten most abundant 16S ESVs identified amongst all samples (*n* = 174).

ESV ID	Phylum	Class	Order	Family	Genus
ESV_5	Bacteroidetes	Bacteroidia	Cytophagales	Microscillaceae	*Chryseolinea*
ESV_6	Proteobacteria	γ-Proteobacteria	Steroidobacterales	Steroidobacteraceae	*Steroidobacter*
ESV_4	Proteobacteria	γ-Proteobacteria	β-Proteobacteriales	Burkholderiaceae	*Massilia*
ESV_3	Proteobacteria	γ-Proteobacteria	β-Proteobacteriales	Burkholderiaceae	*Massilia*
ESV_9	Proteobacteria	α-Proteobacteria	Rhizobiales	Devosiaceae	*Devosia*
ESV_7	Proteobacteria	γ-Proteobacteria	Alteromonadales	Alteromonadaceae	*Rheinheimera*
ESV_13	Bacteroidetes	Bacteroidia	Cytophagales	Microscillaceae	*Chryseolinea*
ESV_11	Proteobacteria	γ-Proteobacteria	Xanthomonadales	Xanthomonadaceae	*Luteimonas*
ESV_12	Bacteroidetes	Bacteroidia	Chitinophagales	Chitinophagaceae	*Parafilimonas*
ESV_8	Proteobacteria	γ-Proteobacteria	Pseudomonadales	Moraxellaceae	*Acinetobacter*
ESV_10	Proteobacteria	γ-Proteobacteria	Pseudomonadales	Pseudomonadaceae	*Pseudomonas*

**Table 4:** Ten most abundant ITS ESVs identified amongst all samples (*n* = 168).

ESV ID	Phylum	Class	Order	Family	Genus
ESV_2	Mortierellomycota	Mortierellomycetes	Mortierellales	Mortierellaceae	*Mortierella*
ESV_4	Ascomycota	Sordariomycetes	Hypocreales	Nectriaceae	*Fusarium*
ESV_3	Mortierellomycota	Mortierellomycetes	Mortierellales	Mortierellaceae	*Mortierella*
ESV_5	Ascomycota	Sordariomycetes	Sordariales	Chaetomiaceae	*Zopfiella*
ESV_11	Ascomycota	Sordariomycetes	Sordariales	Chaetomiaceae	*Zopfiella*
ESV_9	Ascomycota	Sordariomycetes	Microascales	Microascaceae	*Scedosporium*
ESV_13	Mortierellomycota	Mortierellomycetes	Mortierellales	Mortierellaceae	*Mortierella*
ESV_8	Basidiomycota	Unknown	Unknown	Unknown	Unknown
ESV_12	Ascomycota	Eurotiomycetes	Onygenales	Incertae sedis	*Chrysosporium*
ESV_6	Mortierellomycota	Mortierellomycetes	Mortierellales	Mortierellaceae	*Mortierella*
ESV_2	Mortierellomycota	Mortierellomycetes	Mortierellales	Mortierellaceae	*Mortierella*

## Data Availability

Rarified/filtered data, metadata and fasta files for 16S and ITS amplicon sequences are archived for public access at https://doi.org/10.6084/m9.figshare.14731278.v1 (accessed on 21 July 2021), Data are also archived at the University of Colorado Sequencing Facility.

## Supplementary Materials

The following are available online at https://www.mdpi.com/article/10.3390/microorganisms9081561/s1, Table S1: Median percentage of sequences of the most abundant classified bacteria by compost treatment, Table S2: Mean relative abundance of dominant bacterial genera in each root microhabitat, Table S3: Median percentage of sequences of the most abundant classified fungi by compost treatment, Table S4: Mean relative abundance of dominant fungal genera in each root microhabitat, Figure S1: Relationship between gravimetric water content and volumetric water content, Figure S2: Number of unique 16S and ITS ESVs per sample observed for each treatment by microhabitat, Figure S3: Principal coordinate analysis (PCoA) displaying Bray–Curtis dissimilarity of 16S and ITS gene sequences.
