# Supplementary material for: Organic Amendments Alter Soil Hydrology and Belowground Microbiome of Tomato (Solanum lycopersicum)
Source: Microorganisms. 2021 Jul 22;9(8):1561. doi: 10.3390/microorganisms9081561 (PMC8399880; doi:10.3390/microorganisms9081561)
Supplement: Supplementary file 1 [file microorganisms-09-01561-s001.zip › Supplemental InformationV3_clean.pdf]

Supplemental Information

Table S1: Median percentage of sequences of the most abundant classified bacteria by compost treatment[b]

Kingdom[a]	Phylum	Class	Order (-ales)	Family (-aceae)	Genus	UC	VC	DMC	PP
A	Thaumarchaeota	Nitrososphaeria	Nitrososphaer	Nitrososphaer	*Candidatus_Nitrocosmicus*	0.46	0.25	0.18	0.08
B	Acidobacteria	Subgroup_4	Blastocatell	Blastocatell	unknown	0.21	0.14	0.38	0.24
B	Acidobacteria	Subgroup_6	unknown	unknown	unknown	0.42	0.9	0.38	0.24
B	Acidobacteria	Thermoanaerobaculia	Thermoanaerobacul	Thermoanaerobacul	*Subgroup_10*	0	0.37	0	0
B	Actinobacteria	Acidimicrobiia	Actinomarin	unknown	unknown	0	0.84	0	0
B	Actinobacteria	Actinobacteria	Glycomycet	Glycomycet	*Glycomyces*	0	0.04	0.4	0
B	Actinobacteria	Actinobacteria	Micrococc	Promicromonospor	*Isoptericola*	0	0.03	0.45	0.01
B	Actinobacteria	Actinobacteria	Streptosporangi	Thermomonospor	*Actinomadura*	0.23	0.13	0.1	0.09
B	Proteobacteria	α-Proteobacteria	Caulobacter	Caulobacter	*Asticcacaulis*	0.86	0.18	1.12	0.08
B	Proteobacteria	α-Proteobacteria	Caulobacter	Caulobacter	*Brevundimonas*	0.06	0.08	0.26	2.67
B	Proteobacteria	α-Proteobacteria	Caulobacter	Caulobacter	*Phenylobacterium*	0.23	0.08	0.17	0.11
B	Proteobacteria	α-Proteobacteria	Micropeps	Micropeps	*Micropepsis*	0.37	0.11	0.15	0
B	Proteobacteria	α-Proteobacteria	Micropeps	Micropeps	unknown	2.62	0.36	0.46	0.08
B	Proteobacteria	α-Proteobacteria	Rhizobi	Devosi	*Devosia*	1.29	1.19	1.36	0.76
B	Proteobacteria	α-Proteobacteria	Rhizobi	Rhizobi	*Allorhizobium-Neorhizobium-Pararhizobium-Rhizobium*	0.96	0.12	0.5	0.11
B	Proteobacteria	α-Proteobacteria	Rhizobi	Rhizobi	*Brucella*	0.01	0	0.12	0.67
B	Proteobacteria	α-Proteobacteria	Rhizobi	Rhizobi	*Martelella*	0	0	0.2	0.39
B	Proteobacteria	α-Proteobacteria	Rhizobi	Rhizobi	*Mesorhizobium*	0.89	0.12	0.19	0.09
B	Proteobacteria	α-Proteobacteria	Rhizobi	Rhizobi	*Shinella*	0.65	0.55	0.35	0.88
B	Proteobacteria	α-Proteobacteria	Rhizobi	Rhizobi	unknown	0.24	0.17	0.98	0.21
B	Proteobacteria	α-Proteobacteria	Rhizobi	Xanthobacter	*Afipia*	0.48	0.2	0.25	0.04
B	Proteobacteria	α-Proteobacteria	Rhizobi	Xanthobacter	*Bradyrhizobium*	0.49	0.08	0.21	0.01
B	Proteobacteria	α-Proteobacteria	Rhizobi	Xanthobacter	*Pseudolabrys*	1.05	0.37	0.61	0.16
B	Proteobacteria	α-Proteobacteria	Rhizobi	Xanthobacter	*Rhodopseudomonas*	0.44	0.04	0.06	0.01
B	Proteobacteria	α-Proteobacteria	Rhizobi	Xanthobacter	unknown	0.5	0.11	0.15	0.12
B	Proteobacteria	α-Proteobacteria	Rhodobacter	Rhodobacter	unknown	0	0	0.44	0
B	Proteobacteria	α-Proteobacteria	Rhodospirill	Thalassospir	*Thalassospira*	0	0.8	0	0
B	Proteobacteria	α-Proteobacteria	Sphingomonad	Sphingomonad	*Altererythrobacter*	0	0.6	0.54	0.01

B	Proteobacteria	α-Proteobacteria	Sphingomonad	Sphingomonad	*Erythrobacter*	0	0.39	0	0
B	Proteobacteria	α-Proteobacteria	Sphingomonad	Sphingomonad	*Porphyrobacter*	0.29	0.08	0.1	0.01
B	Proteobacteria	α-Proteobacteria	Sphingomonad	Sphingomonad	*Sphingobium*	0.7	0.37	0.16	0.18
B	Proteobacteria	α-Proteobacteria	Sphingomonad	Sphingomonad	*Sphingopyxis*	0.06	0.02	0.61	0.2
B	Bacteroidetes	Bacteroidia	VC2.1_Bac22	unknown	unknown	0	0	0.09	1.13
B	Bacteroidetes	Bacteroidia	Chitinophag	3713	unknown	0.08	0.18	0.09	0.16
B	Bacteroidetes	Bacteroidia	Chitinophag	Chitinophag	*Arachidicoccus*	0.2	0.11	0.04	0.04
B	Bacteroidetes	Bacteroidia	Chitinophag	Chitinophag	*Chitinophaga*	0.1	0	0.02	0.42
B	Bacteroidetes	Bacteroidia	Chitinophag	Chitinophag	*Flavitalea*	0.49	0.15	0.32	0.05
B	Bacteroidetes	Bacteroidia	Chitinophag	Chitinophag	*Niastella*	0.16	0.1	0.17	0.01
B	Bacteroidetes	Bacteroidia	Chitinophag	Chitinophag	*Parafilimonas*	0.85	0.49	0.44	0.21
B	Bacteroidetes	Bacteroidia	Chitinophag	Chitinophag	*Terrimonas*	0.01	0.11	0.35	0.02
B	Bacteroidetes	Bacteroidia	Chitinophag	Chitinophag	unknown	1.58	0.71	0.98	0.54
B	Bacteroidetes	Bacteroidia	Chitinophag	Saprospir	*Membranicola*	0	0.71	0.01	0
B	Bacteroidetes	Bacteroidia	Cytophag	Cyclobacteri	*Algoriphagus*	0	0.31	0.44	0.42
B	Bacteroidetes	Bacteroidia	Cytophag	Cyclobacteri	unknown	0	0	0.13	1.06
B	Bacteroidetes	Bacteroidia	Cytophag	Cytophag	*Cytophaga*	0.19	0	0.33	0
B	Bacteroidetes	Bacteroidia	Cytophag	Cytophag	*Sporocytophaga*	0	0	0.49	0.1
B	Bacteroidetes	Bacteroidia	Cytophag	Microscill	*Chryseolinea*	0.07	7.34	0.3	0.03
B	Bacteroidetes	Bacteroidia	Cytophag	Microscill	unknown	0.35	0.53	0.88	0.36
B	Bacteroidetes	Bacteroidia	Flavobacteri	Crocinitomic	*Fluviicola*	0.42	0.97	0.63	1.35
B	Bacteroidetes	Bacteroidia	Flavobacteri	Cryomorph	*Owenweeksia*	0	1.06	0	0.01
B	Bacteroidetes	Bacteroidia	Flavobacteri	Flavobacteri	*Confluentibacter*	0	0	0.18	1.33
B	Bacteroidetes	Bacteroidia	Flavobacteri	Flavobacteri	*Flavirhabdus*	0	0.9	0	0
B	Bacteroidetes	Bacteroidia	Flavobacteri	Flavobacteri	*Flavobacterium*	0.49	0.59	0.55	0.73
B	Bacteroidetes	Bacteroidia	Flavobacteri	Flavobacteri	*Pricia*	0	0.66	0	0
B	Bacteroidetes	Bacteroidia	Flavobacteri	Flavobacteri	*Subsaxibacter*	0	0.38	0	0
B	Bacteroidetes	Bacteroidia	Flavobacteri	Flavobacteri	*Vitellibacter*	0	0.1	0.56	0.46
B	Bacteroidetes	Bacteroidia	Flavobacteri	Flavobacteri	unknown	0	0.35	0	0
B	Bacteroidetes	Bacteroidia	Flavobacteri	Weeksell	*Chryseobacterium*	0.23	0.07	0	0.04
B	Bacteroidetes	Bacteroidia	Sphingobacteri	env.OPS_17	unknown	1.08	0.11	0.75	0.16
B	Bacteroidetes	Bacteroidia	Sphingobacteri	Sphingobacteri	*Arcticibacter*	0.09	0.01	0.02	1.53

B	Bacteroidetes	Bacteroidia	Sphingobacteri	Sphingobacteri	*Mucilaginibacter*	1.06	0	0.02	0
B	Bacteroidetes	Bacteroidia	Sphingobacteri	Sphingobacteri	*Olivibacter*	0	0	0.48	0
B	Bacteroidetes	Bacteroidia	Sphingobacteri	Sphingobacteri	*Parapedobacter*	0	0.02	0.96	0.19
B	Bacteroidetes	Bacteroidia	Sphingobacteri	Sphingobacteri	*Pedobacter*	0.54	0.93	0.19	2.81
B	Bacteroidetes	Bacteroidia	Sphingobacteri	Sphingobacteri	*Pelobium*	0.07	0.33	0.02	0.29
B	Bacteroidetes	Bacteroidia	Sphingobacteri	Sphingobacteri	*Sphingobacterium*	0.04	0.01	1.72	1.6
B	Bacteroidetes	Bacteroidia	Sphingobacteri	Sphingobacteri	unknown	0.01	0.36	0	0.2
B	Bacteroidetes	Ignavibacteria	Ignavibacteri	unknown	unknown	0	0.66	0	0
B	Bacteroidetes	Rhodothermia	Rhodotherm	Rhodotherm	unknown	0	1.39	0	0.01
B	Chloroflexi	Anaerolineae	Anaeroline	Anaeroline	unknown	0.76	0.36	0.23	0.21
B	Chloroflexi	Anaerolineae	SBR1031	unknown	unknown	0.29	0.16	0.11	0.08
B	Proteobacteria	δ-Proteobacteria	Myxococc	BIrii41	unknown	0	0.25	0.79	0
B	Proteobacteria	δ-Proteobacteria	Myxococc	Nannocyst	unknown	0	0	0.47	0
B	Proteobacteria	δ-Proteobacteria	NB1j	unknown	unknown	0.3	0.16	0.1	0.08
B	Firmicutes	Bacilli	Bacill	Bacill	*Bacillus*	1.06	0.28	0.27	0.1
B	Firmicutes	Bacilli	Bacill	Planococc	unknown	0.77	0.32	0.23	0.7
B	Proteobacteria	γ-Proteobacteria	Alteromonad	Alteromonad	*Rheinheimera*	0.03	2.72	1.04	0.42
B	Proteobacteria	γ-Proteobacteria	Alteromonad	Alteromonad		0	0.37	0	0
B	Proteobacteria	γ-Proteobacteria	Betaproteobacteri	Burkholderi	*Achromobacter*	0.22	0.06	0.27	1.53
B	Proteobacteria	γ-Proteobacteria	Betaproteobacteri	Burkholderi	*Comamonas*	0.18	0	0.01	0.38
B	Proteobacteria	γ-Proteobacteria	Betaproteobacteri	Burkholderi	*Massilia*	7.84	0.13	0.19	0.16
B	Proteobacteria	γ-Proteobacteria	Betaproteobacteri	Burkholderi	*Noviherbaspirillum*	0.73	0.22	0.34	0.01
B	Proteobacteria	γ-Proteobacteria	Betaproteobacteri	Burkholderi	*Ramlibacter*	0.29	0.13	0.24	0.05
B	Proteobacteria	γ-Proteobacteria	Betaproteobacteri	Burkholderi	unknown	1.16	0.16	0.07	0.02
B	Proteobacteria	γ-Proteobacteria	Betaproteobacteri	Methylophil	*Methylobacillus*	0.27	0.07	0.01	0
B	Proteobacteria	γ-Proteobacteria	Betaproteobacteri	Methylophil	*Methylophilus*	0.19	0.06	0.12	0.05
B	Proteobacteria	γ-Proteobacteria	Betaproteobacteri	Nitrosomonad	*Ellin6067*	0.49	0.19	0.22	0.05
B	Proteobacteria	γ-Proteobacteria	Betaproteobacteri	Rhodocycl	*Uliginosibacterium*	0.15	0.13	0.17	0.01
B	Proteobacteria	γ-Proteobacteria	CCD24	unknown	unknown	0	0.56	0	0
B	Proteobacteria	γ-Proteobacteria	Cellvibrion	Cellvibrion	*Cellvibrio*	0.03	1.29	1.29	0.32
B	Proteobacteria	γ-Proteobacteria	Cellvibrion	Cellvibrion		0.39	0.78	0.22	0.51
B	Proteobacteria	γ-Proteobacteria	Diplorickettsi	Diplorickettsi	*Aquicella*	0.33	0.01	0.01	0

B	Proteobacteria	γ-Proteobacteria	Enterobacteri	Enterobacteri	*Klebsiella*	0.47	0.01	0.02	1.36
B	Proteobacteria	γ-Proteobacteria	Incertae_Sedis	unknown	*Acidibacter*	0.28	0.08	0.14	0
B	Proteobacteria	γ-Proteobacteria	Oceanospirill	Halomonad	*Halomonas*	0	0.34	0.02	0
B	Proteobacteria	γ-Proteobacteria	Oceanospirill	Saccharospirill	*Saccharospirillum*	0	0.42	0	0
B	Proteobacteria	γ-Proteobacteria	Pseudomonad	Moraxell	*Acinetobacter*	0.29	0.07	0.24	4.23
B	Proteobacteria	γ-Proteobacteria	Pseudomonad	Pseudomonad	*Pseudomonas*	2.55	3.87	2.6	7.2
B	Proteobacteria	γ-Proteobacteria	Steroidobacter	Steroidobacter	*Steroidobacter*	0.71	0.43	2.94	0.55
B	Proteobacteria	γ-Proteobacteria	Xanthomonad	Rhodanobacter	*Dokdonella*	0.5	0.25	0.24	0.14
B	Proteobacteria	γ-Proteobacteria	Xanthomonad	Rhodanobacter	*Dyella*	0.22	0.06	0.06	0.05
B	Proteobacteria	γ-Proteobacteria	Xanthomonad	Rhodanobacter	*Rhodanobacter*	1.49	0.99	1.21	1.93
B	Proteobacteria	γ-Proteobacteria	Xanthomonad	Xanthomonad	*Luteimonas*	0.68	1.84	1.71	0.46
B	Proteobacteria	γ-Proteobacteria	Xanthomonad	Xanthomonad	*Lysobacter*	0	0.61	0	0
B	Proteobacteria	γ-Proteobacteria	Xanthomonad	Xanthomonad	*Stenotrophomonas*	0.01	0.03	0.72	1.41
B	Proteobacteria	γ-Proteobacteria	Xanthomonad	Xanthomonad	*Thermomonas*	0.42	0.26	0.09	0.31
B	Proteobacteria	γ-Proteobacteria	unknown	unknown	unknown	0	0.35	0	0
B	Gemmatimonadetes	Gemmatimonadetes	Gemmatimonad	Gemmatimonad	unknown	0.45	0.17	0.34	0.06
B	Gemmatimonadetes	S0134	unknown	unknown	unknown	0.08	0.77	0.31	0.03
B	Planctomycetes	Planctomycetacia	Pirellul	Pirellul	*Pirellula*	0.27	0.06	0.11	0.08
B	Planctomycetes	Planctomycetacia	Pirellul	Pirellul	*Rhodopirellula*	0.19	0.02	0.06	0.18
B	Planctomycetes	Planctomycetacia	Pirellul	Pirellul	*Roseimaritima*	0	0	0.22	2.11
B	Planctomycetes	Planctomycetacia	Planctomycet	unknown	unknown	0.14	0.04	0.09	0.14
B	Verrucomicrobia	Verrucomicrobiae	Pedosphaer	Pedosphaer	unknown	0.26	0.2	0.47	0.36
B	Verrucomicrobia	Verrucomicrobiae	Verrucomicrobi	Rubritale	*Luteolibacter*	0.19	0.52	0.66	0.63

[a]A: Archaea, B: Bacteria

[b]: UC: untreated (conventionally fertilized) control, DMC: dairy manure compost, VC: vermicompost, PP: poultry pellets

Table S2. Mean relative abundance of dominant bacterial genera in each root microhabitat. Bolded numbers represent the greatest value observed for each genus. Filtered to exclude genera which did not account for 0.5% of the bacterial community. Data presented represents pooled samples from all treatments and harvest times (n = 174).

Genus	Bulk soil	Rhizosphere	Rhizoplane	Endosphere (within root)
Acinetobacter	**1.94%**	1.37%	0.62%	0.63%
Allorhizobium-Neorhizobium-Pararhizobium-Rhizobium	0.14%	0.39%	1.53%	**3.09%**
Asticcacaulis	0.60%	0.77%	1.60%	**2.26%**
Bacillus	0.42%	**1.75%**	0.40%	0.72%
Cellvibrio	0.93%	0.90%	**2.51%**	2.25%
Chryseolinea	4.99%	**5.82%**	2.10%	1.35%
Devosia	2.04%	1.89%	1.89%	**4.06%**
Flavobacterium	2.16%	1.83%	**2.54%**	2.28%
Fluviicola	2.23%	0.83%	1.72%	**2.77%**
Luteimonas	2.83%	**3.09%**	2.49%	1.47%
Massilia	1.64%	1.51%	**7.89%**	7.37%
Pedobacter	**3.48%**	2.01%	2.78%	3.27%
Pseudolabrys	1.63%	**1.69%**	0.47%	0.46%
Pseudomonas	2.84%	5.95%	**8.77%**	8.32%
Rheinheimera	0.36%	0.55%	**4.73%**	3.31%
Rhodanobacter	**2.45%**	2.17%	1.88%	1.46%
Shinella	0.26%	0.52%	1.00%	**2.73%**
Sphingobacterium	**2.16%**	1.15%	0.92%	0.77%
Stenotrophomonas	0.34%	0.51%	1.65%	**1.92%**
Steroidobacter	2.50%	**2.96%**	1.74%	0.59%
Other	64.05%	62.34%	50.78%	48.94%

Table S3: Median percentage of sequences of the most abundant classified fungi by compost treatment[a].

Phylum (-mycota)	Class (-cetes)	Order (-ales)	Family (-aceae)	Genus	UC	V	DMC	PP
Asco	Eurotiomy	Euroti	Aspergill	*Aspergillus*	0.43	0.09	0.52	0.05
Asco	Eurotiomy	Euroti	Aspergill	*Penicillium*	0.42	0	0	0
Asco	Eurotiomy	Euroti	Aspergill	*Phialosimplex*	1.25	0	0	0
Asco	Eurotiomy	Euroti	Trichocom	*Thermomyces*	0	0.16	0.36	0
Asco	Eurotiomy	Onygen	Incertae_sedis	*Chrysosporium*	4.06	4.1	2.61	1.08
Asco	Eurotiomy	unknown	unknown	unknown	0	0.95	0.21	0
Asco	Leotiomy	Thelebol	Pseudeuroti	*Gymnostellatospora*	1.08	3.13	0.73	0.41
Asco	Leotiomy	Thelebol	Pseudeuroti	*Pseudogymnoascus*	0	0.29	0	0
Asco	Orbiliomy	Orbili	Orbili	*Arthrobotrys*	0.24	0.03	1.18	10.56
Asco	Pezizomy	Peziz	Ascobol	*Ascobolus*	0	0.17	0	1.29
Asco	Pezizomy	Peziz	Ascobol	unknown	0	0	0.39	5.19
Asco	Pezizomy	Peziz	Ascodesmid	*Cephaliophora*	0	0	0	4.04
Asco	Pezizomy	Peziz	Peziz	*Iodophanus*	3.45	0	2.5	4.61
Asco	Pezizomy	Peziz	Peziz	unknown	0.08	0.06	0	0
Asco	Pezizomy	Peziz	Pyronemat	*Scutellinia*	0	0	0	0.96
Asco	Pezizomy	Peziz	unknown	unknown	0	0	0	0.88
Asco	Pezizomy	unknown	unknown	unknown	0	0	0.08	0.35
Asco	Saccharomy	Saccharomycet	Debaryomycet	*Debaryomyces*	0	0.09	0	0
Asco	Saccharomy	Saccharomycet	Debaryomycet	*Meyerozyma*	0	0	0	0.22
Asco	Saccharomy	Saccharomycet	Saccharomycet	*Zygosaccharomyces*	0.3	0.06	0	0
Asco	Saccharomy	Saccharomycet	Incertae_sedis	*Candida*	0.65	0.59	0.59	0.55
Asco	Sordariomy	Glomerell	Plectosphaerell	*Sodiomyces*	0	0.14	0	0
Asco	Sordariomy	Glomerell	unknown	unknown	0	0	0.33	0
Asco	Sordariomy	Hypocre	Cordycipit	*Engyodontium*	0	0.13	0	0
Asco	Sordariomy	Hypocre	Cordycipit	*Lecanicillium*	0	0.08	0	0
Asco	Sordariomy	Hypocre	Hypocre	*Trichoderma*	0.31	0.05	0.03	0.01
Asco	Sordariomy	Hypocre	Incertae_sedis	*Sarocladium*	0.02	0	0.1	0
Asco	Sordariomy	Hypocre	Nectri	*Fusarium*	21.64	10.67	0.65	1.99
Asco	Sordariomy	Hypocre	Nectri	*Gibberella*	0	0.17	0	0
Asco	Sordariomy	Hypocre	Stachybotry	*Stachybotrys*	0	0.11	0.1	0
Asco	Sordariomy	Hypocre	unknown	unknown	0	0	0.12	0
Asco	Sordariomy	Microasc	Halosphaeri	*Cirrenalia*	0.07	0.1	0.18	0.03
Asco	Sordariomy	Microasc	Microasc	*Pseudallescheria*	0.01	0.3	0.03	0.14
Asco	Sordariomy	Microasc	Microasc	*Scedosporium*	0	1.71	27.55	0.31
Asco	Sordariomy	Microasc	Microasc	unknown	0.01	3.04	1.11	0
Asco	Sordariomy	Microasc	unknown	unknown	0	1.18	0.85	0
Asco	Sordariomy	Sordari	Chaetomi	*Botryotrichum*	0.01	3.5	1.1	1.02
Asco	Sordariomy	Sordari	Chaetomi	*Chaetomium*	1.01	0.63	0.3	0.16
Asco	Sordariomy	Sordari	Chaetomi	*Humicola*	0	0.01	0.02	0.87
Asco	Sordariomy	Sordari	Chaetomi	*Myceliophthora*	0	0.3	0.04	0

Asco	Sordariomy	Sordari	Chaetomi	*Mycothermus*	0.01	0.75	0.44	0.01
Asco	Sordariomy	Sordari	Chaetomi	*Zopfiella*	14.35	9.09	22.15	1.29
Asco	Sordariomy	Sordari	Chaetomi	unknown	0.43	0	0	0.74
Asco	Sordariomy	Sordari	Lasiosphaeri	unknown	4.01	9.89	1.35	0.05
Asco	Sordariomy	Sordari	unknown	unknown	2.07	0.32	1.15	0
Asco	Sordariomy	Xylari	Xylari	unknown	0	0.11	0.02	0
Asco	Sordariomy	unknown	unknown	unknown	0.38	0.59	0.35	0.18
Asco	unknown	unknown	unknown	unknown	0.28	1.48	0.46	0.26
Basidio	Agaricomy	Polypor	Fomitopsid	*Piptoporus*	0.11	0.23	0.12	0
Basidio	Agaricomy	Sebacin	Serendipit	*Serendipita*	0.17	0	0	0
Basidio	Agaricomy	unknown	unknown	unknown	1.57	1.34	0.4	0.5
Basidio	Tremellomy	Filobasidi	Filobasidi	*Filobasidium*	0.04	1.08	0	0.01
Basidio	Tremellomy	Filobasidi	Filobasidi	*Naganishia*	0	0.09	0	0
Basidio	Tremellomy	Filobasidi	Piskurozym	*Solicoccozyma*	0.25	0	0	0
Basidio	Tremellomy	Tremell	Tremell	*Bulleromyces*	0	0.26	0	0
Basidio	Tremellomy	Trichosporon	Trichosporon	*Cutaneotrichosporon*	0.75	0	0	0
Basidio	unknown	unknown	unknown	unknown	3.15	11.08	14.93	8.21
Mortierello	Mortierellomy	Mortierell	Mortierell	*Mortierella*	34.44	26.95	12.86	50.83
Rozello	unknown	unknown	unknown	unknown	0	1.03	0	0

[a]: UC: untreated (conventionally fertilized) control, DMC: dairy manure compost, VC: vermicompost, PP: poultry pellets

Table S4 Mean relative abundance of dominant fungal genera in each root microhabitat. Bolded numbers represent the greatest value observed for each genus. Filtered to exclude genera which did not account for 0.5% of the fungal community. Data presented represents pooled samples from all treatments and harvest times (n = 168). Empty cells for taxonomy indicate the absence of resolution of identification at that level.

**Phylum (-mycota)**	**Class (-cetes)**	**Order (-ales)**	**Family (-aceae)**	**Genus**	**Bulk soil**	**Rhizosphere**	**Rhizoplane**	**Endosphere (within root)**
Asco	Eurotiomy	Onygen	Incertae_sedis	*Chrysosporium*	4.0%	3.5%	2.6%	2.5%
Asco	Leotiomy	Thelebol	Pseudeuroti	*Gymnostellatospora*	1.4%	2.1%	1.7%	0.3%
Asco	Orbiliomy	Orbili	Orbili	*Arthrobotrys*	2.7%	1.7%	3.4%	1.6%
Asco	Pezizomy	Peziz	Ascobol	unknown	2.1%	0.4%	0.1%	0.9%
Asco	Pezizomy	Peziz	Ascodesmid	*Cephaliophora*	2.2%	0.0%	0.0%	0.0%
Asco	Pezizomy	Peziz	Peziz	*Iodophanus*	4.8%	2.0%	0.0%	0.3%
Asco	Saccharomy	Saccharomycet	Incertae_sedis	*Candida*	1.1%	0.3%	0.8%	0.4%
Asco	Sordariomy	Hypocre	Nectri	*Fusarium*	3.2%	4.8%	18.0%	**31.1%**
Asco	Sordariomy	Microasc	Microasc	unknown	1.5%	1.5%	0.6%	0.9%
Asco	Sordariomy	Microasc	Microasc	*Scedosporium*	6.3%	7.4%	9.3%	7.0%
Asco	Sordariomy	Microasc	unknown	unknown	0.3%	0.6%	1.8%	0.3%
Asco	Sordariomy	Sordari	Chaetomi	*Botryotrichum*	1.2%	2.1%	1.8%	0.7%
Asco	Sordariomy	Sordari	Chaetomi	*Chaetomium*	0.8%	0.8%	0.0%	0.2%
Asco	Sordariomy	Sordari	Chaetomi	*Zopfiella*	10.1%	17.1%	8.1%	8.0%
Asco	Sordariomy	Sordari	Lasiosphaeri	unknown	3.0%	7.1%	2.8%	2.3%
Asco	Sordariomy	Sordari	unknown	unknown	0.1%	1.6%	1.3%	0.9%
Asco	unknown	unknown	unknown	unknown	0.6%	0.8%	2.5%	0.2%
Basidio	Agaricomy	unknown	unknown	unknown	2.3%	0.7%	0.7%	1.1%
Basidio	unknown	unknown	unknown	unknown	12.3%	8.3%	10.5%	11.6%
Mortierello	Mortierellomy	Mortierell	Mortierell	*Mortierella*	**34.2%**	**30.4%**	**25.7%**	25.5%
Other					5.6%	6.9%	8.4%	4.2%

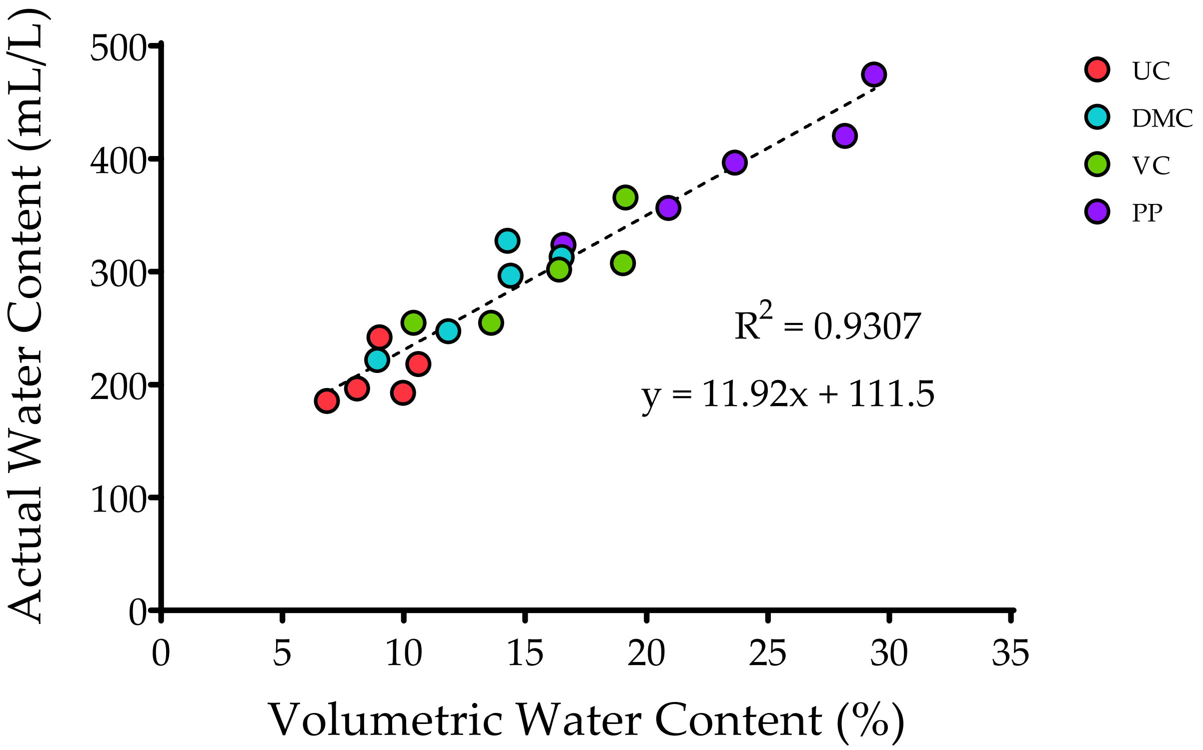

Figure S1. Relationship between gravimetric water content (*y*-axis) and volumetric water content (*x*-axis). Points represent means (n = 3) at decreasing matric potentials (40, 100, 200, 300, 500 kPa). Treatments represent soil with or without amendment of vermicompost (VC, 20% v/v), dairy manure compost (DMC, 20% v/v), or dehydrated poultry manure pellets (PP, 10% v/v) in comparison to an untreated (conventionally fertilized) control (UC).

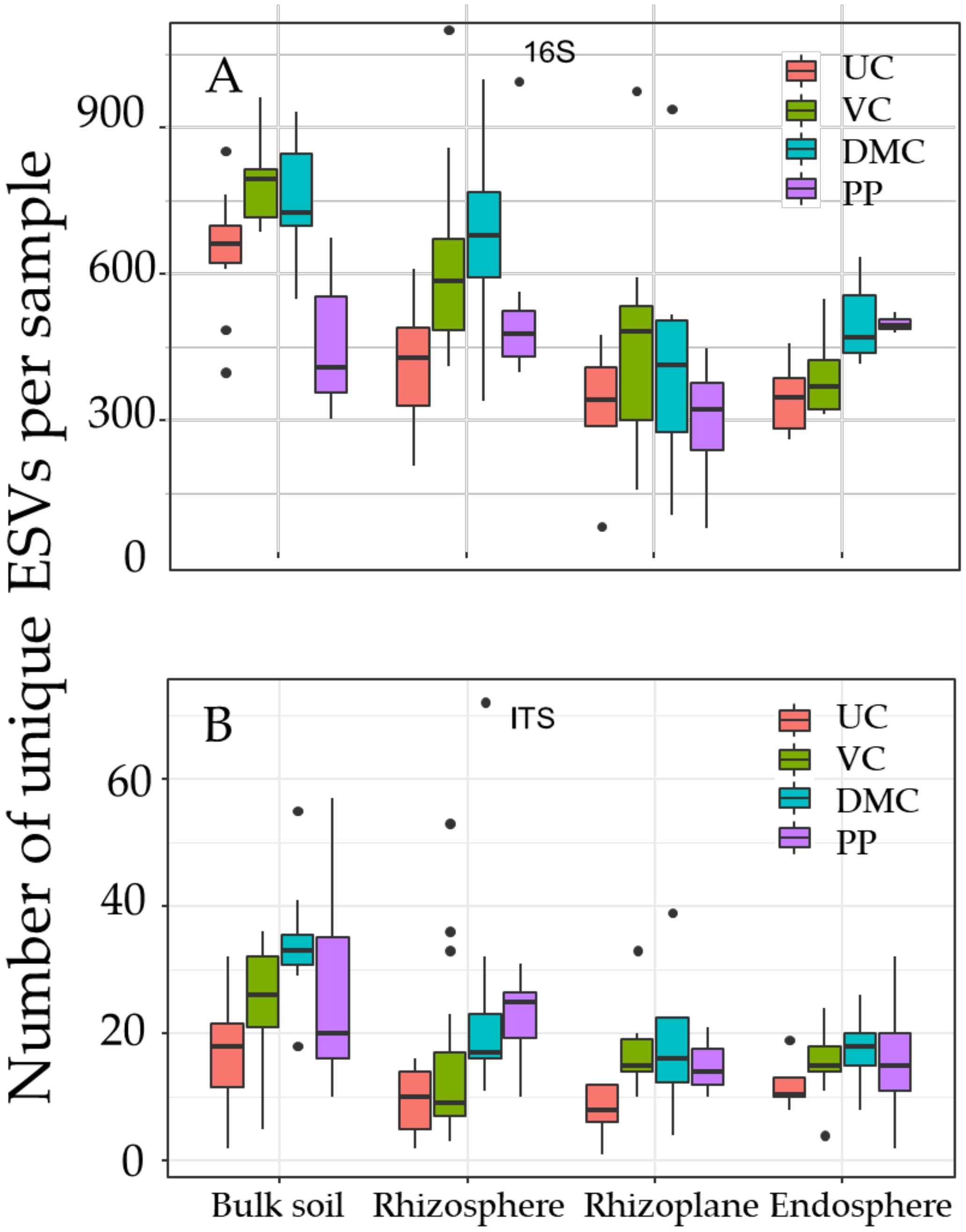

Figure S2. Number of unique ESVs per sample observed for each treatment by microhabitat. Illustrated are box plots for A) 16S rRNA (n = 174) and B) ITS (n = 168) amplicon sequences. Treatments represent growing mix and soil with or without amendment of vermicompost (VC, 20% v/v), dairy manure compost (DMC, 20% v/v), or dehydrated poultry manure pellets (PP, 10% v/v) compared to an untreated (conventionally fertilized) control (UC). Data presented represent pooled samples from all harvest times.

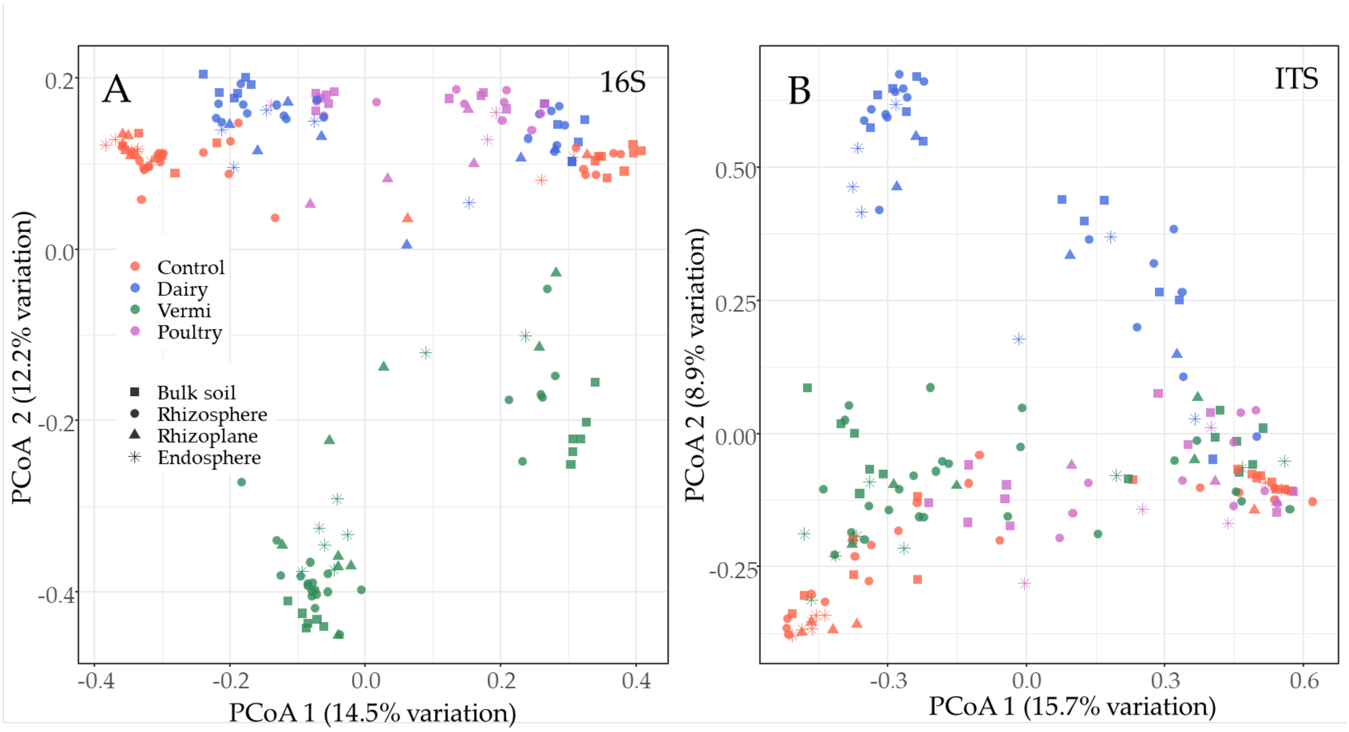

Figure S3. Principal Coordinates Analysis (PCoA) biplot displaying Bray Curtis dissimilarity of A) 16S (n = 174) and B) ITS (n = 168) amplicon sequences among all samples in complete dataset. Treatments represent growing mix and soil with amendment of vermicompost (green, 20% v/v), dairy manure compost (blue, 20% v/v), or dehydrated poultry manure pellets (purple, 10% v/v) compared to an untreated (conventionally fertilized) control (red). Symbol shape represents microhabitat: square = bulk soil, circle = rhizosphere, triangle = rhizoplane, star = endosphere.
