# Supplementary figures and images for: Organic Amendments Alter Soil Hydrology and Belowground Microbiome of Tomato (Solanum lycopersicum)
Source: Microorganisms. 2021 Jul 22;9(8):1561. doi: 10.3390/microorganisms9081561 (PMC8399880; doi:10.3390/microorganisms9081561)

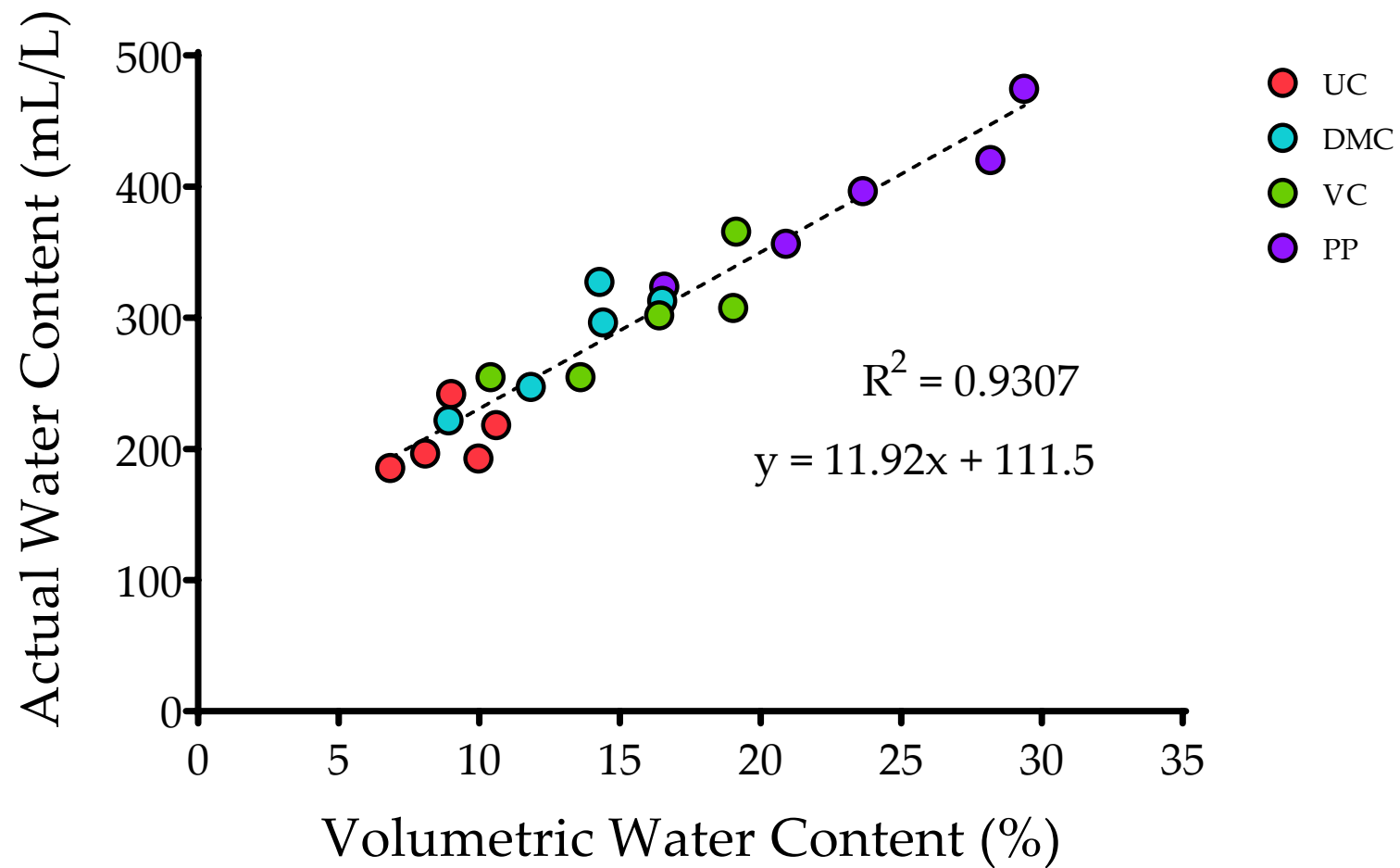

Supplement: Supplementary file 1 [file microorganisms-09-01561-s001.zip › Figure S1.pdf]

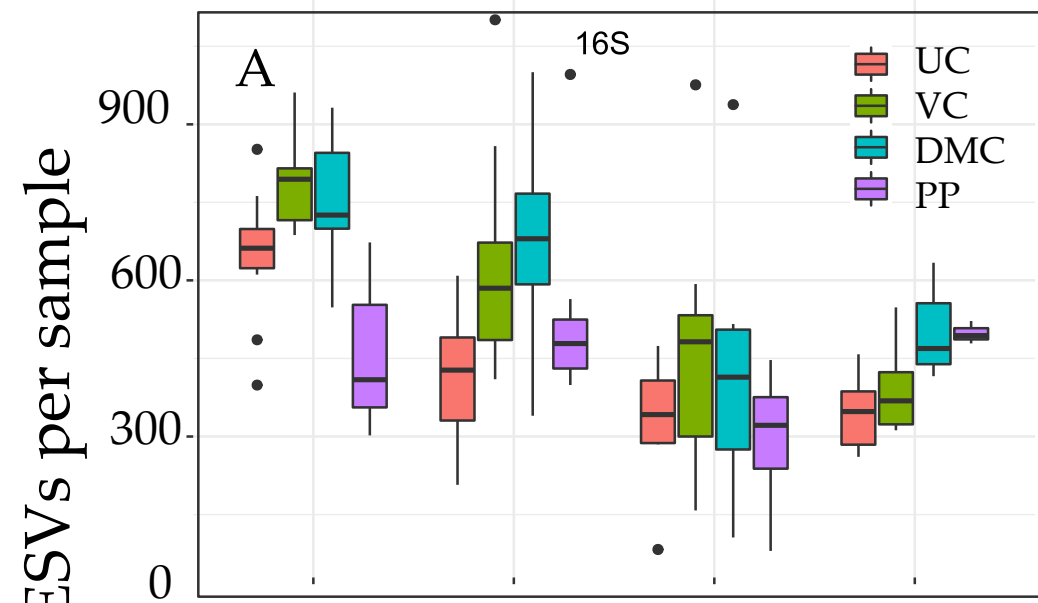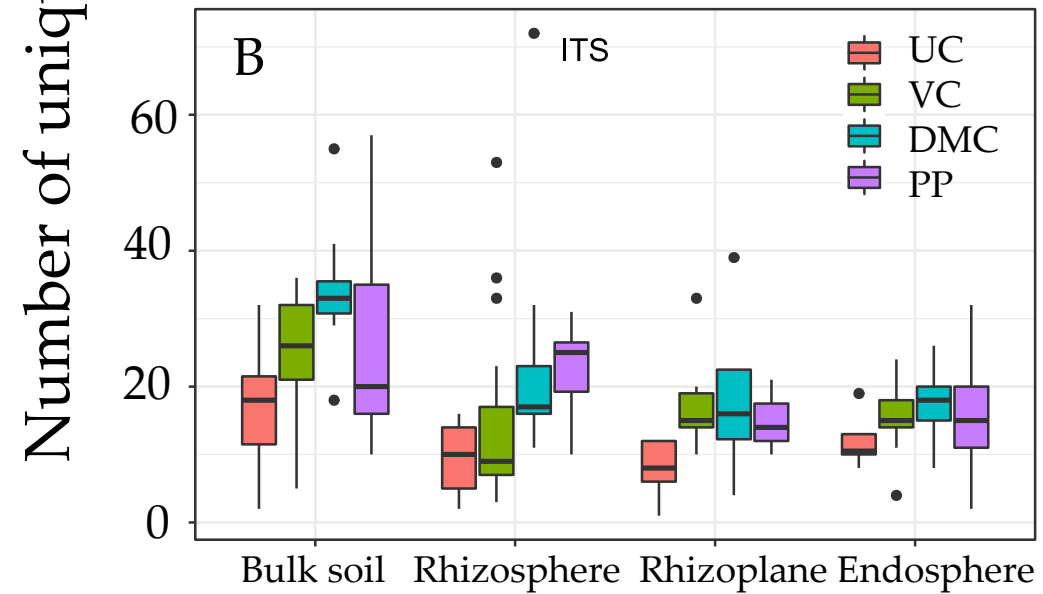

Microhabitat

Supplement: Supplementary file 1 [file microorganisms-09-01561-s001.zip › Figure S2.pdf]

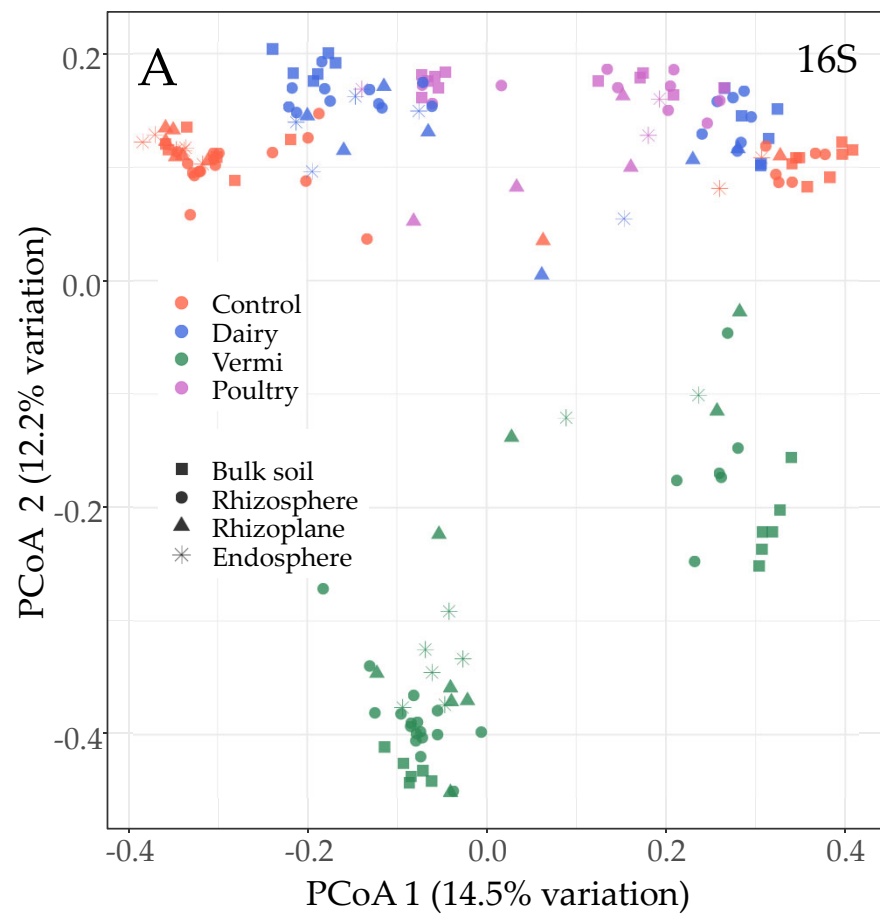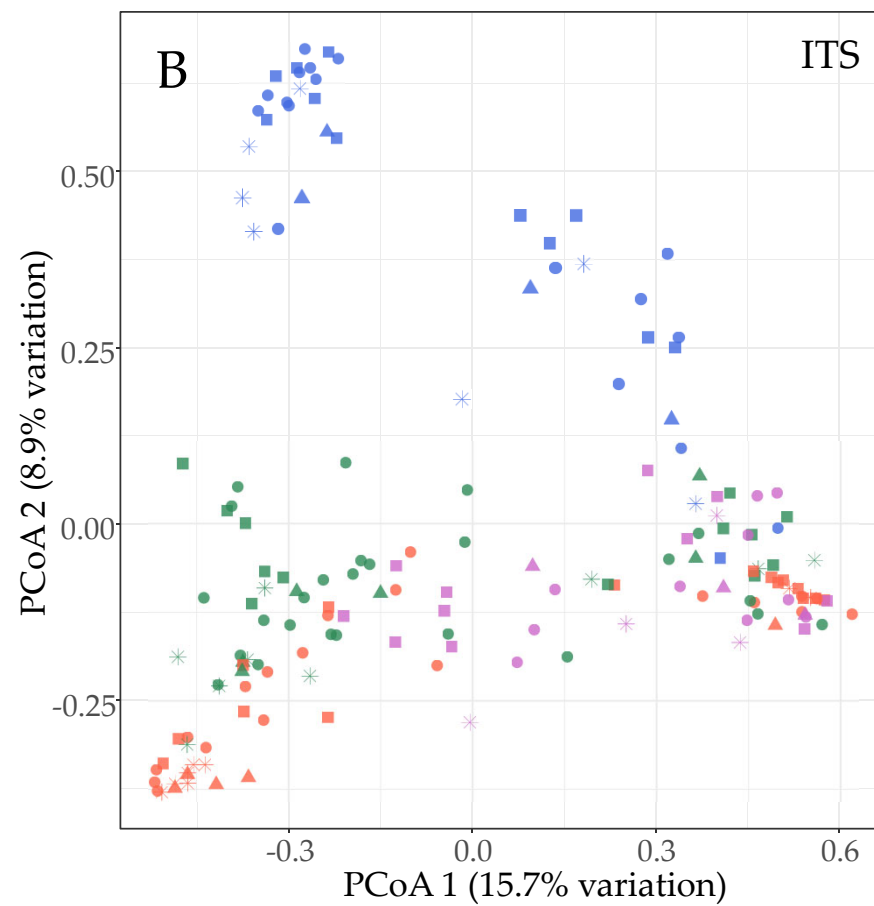

Supplement: Supplementary file 1 [file microorganisms-09-01561-s001.zip › Figure S3.pdf]
